# *Panax notoginseng* saponins modulate the gut microbiota to promote thermogenesis and beige adipocyte reconstruction *via* leptin-mediated AMPKα/STAT3 signaling in diet-induced obesity

**DOI:** 10.7150/thno.47746

**Published:** 2020-09-14

**Authors:** Yu Xu, Ning Wang, Hor-Yue Tan, Sha Li, Cheng Zhang, Zhangjin Zhang, Yibin Feng

**Affiliations:** School of Chinese Medicine, Li Ka Shing Faculty of Medicine, The University of Hong Kong.

**Keywords:** Gut microbiota, Obesity, White adipocyte, Beige cell, Leptin

## Abstract

**Background:** Activation of the thermogenic program in white and brown adipocytes presents a promising avenue for increasing energy expenditure during the treatment of obesity. The endogenous mechanism for promoting thermogenesis in brown adipocytes or browning in white adipocytes has indicated that the gut microbiota is a crucial regulator of the host energy balance. However, whether the effects of the therapeutic intervention-induced modulation of the gut microbiota on adipocyte browning involved the regulation of leptin remains unclear.

**Method:** The adipose features were analyzed by body composition analysis, infrared camera observations, transmission electron microscopy and H&E staining. The gene and protein expression in adipose tissue were detected by qRT-PCR, immunoblotting, immunohistochemistry and immunofluorescence staining. The gut microbiome signature was identified by 16S rRNA gene amplicon sequencing, and both mice with high-fat diet-induced obesity (DIO) and mice with antibiotics-induced microbiome depletion were subjected to fecal microbiota transplantation.

**Results:** Treatment with *Panax notoginseng* saponins (PNS) shaped the murine gut microbiome by increasing the abundances of *Akkermansia muciniphila* and *Parabacteroides distasonis*, and as a result, DIO mice harbored a distal gut microbiota with a significantly increased capacity to reduce host adiposity. The PNS-induced modulation of the gut microbiota in DIO mice could increase brown adipose tissue (BAT) thermogenesis and beige adipocyte reconstruction by activating the leptin-AMPK/STAT3 signaling pathway, which results in the promotion of energy expenditure. Leptin has an essential influence on the anti-obesity effects of PNS. In cases of leptin deficiency, the PNS-induced modulation of the gut microbiota exerts negative effects on thermogenesis and browning in white adipose tissue (WAT), which indicates that PNS fail to reduce obesity in leptin gene-deficient mice. The PNS-induced modulation of the gut microbiota exerted a minimal effect on DIO mice with antibiotic-induced microbiome depletion, which confirmed the correlation between altered gut microbiota and the remodeling of adipose tissues in DIO mice. The direct influence of leptin on browning *via* the AMPKα/STAT3 signaling pathway in C3H101/2 cells supported our *in vivo* results that signalling through the leptin-AMPK/STAT3 pathway induced by the PNS-modulated gut microbiota was involved in beige adipocyte reconstruction.

**Conclusion**: Our results revealed that leptin signaling is critical for alterations in microbiota-fat crosstalk and provide promising avenues for therapeutic intervention in the treatment of obesity.

## Introduction

Obesity, which is one of the most prevalent global epidemics, is a result of excessive adipocyte accumulation 1, and fat build-up might contribute to an imbalance between calorie intake and energy expenditure. Adipocytes of different types have distinct opposite functions in regulating energy homeostasis. Brown adipose tissue (BAT) dissipates energy through the activation of uncoupling protein 1 (UCP-1) found in mitochondria and converts fat through thermogenesis to defend against obesity. In contrast, white adipose tissue (WAT) is responsible for energy storage *via* the synthesis and accumulation of triglycerides 2. Epididymal adipocyte accumulation is closely correlated with the development of obesity and insulin resistance. In contrast, the expansion of subcutaneous adipocytes shows little or even an inverse correlation with the risk of obesity 3. Other thermogenic cells in WAT are defined as beige or brown-like adipocytes 4, which exhibit increased thermogenic abilities and similar features to brown adipocytes 5 and can be converted from other cells in WAT 6 through the expression of UCP-1, peroxisome proliferator-activated receptor-γ coactivator 1-α (PGC1-α), and PR domain-containing 16 (PRDM-16) 7.

Accumulating data show that the gut microbiota is a vital endogenous factor in regulating white-to-brown fat conversion and non-shivering thermogenesis in BAT 8. Previous studies showed that the transfer of “cold” microbiota could increase the rate of beige adipocyte reconstruction 9 in recipients and consecutively improves insulin sensitivity and energy consumption. The WAT of mice with antibiotic-induced microbiome depletion exhibits a browning phenotype, and microbiota depletion promotes eosinophil infiltration, type 2 cytokine activation and M2 macrophage polarization, which results in beige cell reconstruction 10. Intermittent fasting-induced alteration in the gut microbiota can stimulate the expression of monocarboxylate transporter 1 in beige cells of DIO mice 11 but has negative effects on thermogenesis in genetically (ob/ob) obese mice 12. Leptin, as a secretory protein of adipocytes, is a mediator in the regulation of energy intake and consumption and can increase BAT thermogenesis *via* its action on the hypothalamus 13, as well as enhance the browning of WAT by activating the Janus-activated kinase 2 (JAK2)/signal transducer and activator of transcription 3 (STAT3) pathway 14 or inducing the STAT3-PRDM 16 complex 15. The gut microbiota can influence leptin-associated pathways to alleviate obesity-related metabolic disorders, and the action of the gut microbiota on the leptin expression and body weight can be affected by dietary fat intake 16. Thus, researchers will conduct more detailed studies on the anti-obesity effects of nutritional factors targeting the gut microbiota on the participation of leptin in browning. From this perspective, we hypothesize that dietary herb treatment would be able to promote browning by regulating the abundance of different species comprising the gut microbiota in obese mice and that these effects are mediated *via* leptin signalling. Preliminary studies have shown that a commercial herb product of *Panax notoginseng* saponins (PNS), which includes ginsenosides Rb1, Rd, Re, Rf and Rg1 and notoginsenoside R1 (Figure S1A), could control the body weight of obese mice 17. PNS exert lipid-lowering effects by improving the biosynthesis of bile acids from cholesterol and increasing hepatic fatty acid β-oxidation 18, and its antioxidative and anti-inflammatory effects are involved in its anti-hyperglycemic effects in diabetic mice 19, 20. The gut microbiota has important influences based on its activation of metabolites and pharmacological activities 21, which have been extensively investigated 22. PNS have low drug permeability^23^, which results in poor intestinal absorption into the human body, and are thus able to come into contact with the gut microflora for a longer time 23, 24. Thus, PNS with a longer residual time in the intestinal tract might be able to influence the gut microbial ecosystem. In this study, we found that PNS could decrease adiposity *via* BAT thermogenesis and beige adipocyte reconstruction and that PNS modulate the gut microbiota to induce beige adipocyte reconstruction by activating signaling through the leptin-AMPK/STAT3 pathway in DIO mice. This study offers another novel example in which changes in the gut microbiota lead to leptin activation and thereby induce browning remodelling in WAT and indicates that PNS can potentially be used as modulators of the gut microbiota to induce adipose remodelling *via* leptin activation and thereby combat obesity and related metabolic disorder.

## Methods

### UPLC analysis of the medicinal product PNS

PNS derived from *Panax notoginsen*g were purchased from China. An ultra-high-performance liquid chromatography (UHPLC) system (Dionex UltiMate® 3000, Thermo Fisher Scientific, Dreieich, Germany) was used to identify and confirm the percentages of saponins, including notoginsenoside R1 and ginsenosides Rg1, Re, Rf, Rb1 and Rd, in PNS. The separation was performed using a reverse-phase ACE Excel C18 (100 mm × 2.1 mm) analytical column. For the UHPLC analysis, 5 μl of the PNS samples were injected with a flow rate of 0.5 ml/min. Modified gradient conditions with acetonitrile and water were used for the analysis of the samples. Gradient elution was started with 20% acetonitrile for 10 min, and the concentration of acetonitrile was increased from 20 to 46% over 15 min, increased from 46% to 55% over 15 min and maintained at 55% for 5min. The detection wavelength was 203 nm. Feces from the high-fat diet (HFD)+PNS group were collected, dissolved into menthol and prepared for UHPLC analysis using the protocol as mentioned above. PNS mainly contained notoginsenoside R1 and ginsenosides Rb1, Rd, Re, Rf, and Rg1, which have a similar chemical structure as triterpenoid saponin, as shown in Figure S1A. We determined that PNS comprised 9.76% notoginsenoside R1, 35.1% Rg1, and 38.98% Rb1 25.

### Animal experiments

All the animal experiments were approved by the Committee on the Use of Live Animals in Teaching and Research (CULATR) of the University of Hong Kong. Obese B6. Cg-Lep ^ob^/J (ob/ob) and C57BLKS/J-Lepr^db^/J (db/db) male mice fed a regular diet were purchased from The Jackson Laboratory (Bar Harbor, ME). Four-weeks-old male wild-type C57BL/6J mice were fed a HFD (60% fat, 20% protein, 20% carbohydrate; Research Diets, D12492) to induce and maintain obesity. These mice were fed the HFD for 2 weeks at the beginning of the experiment. When an apparent change between the regular chow diet-fed control group and the HFD-fed group was observed, PNS solution (400 or 800 mg/kg per day) or vehicle was orally administered to the HFD-fed mice for 7 weeks. At 6-9 weeks of age, the db/db and ob/ob mice received injections of the PNS solution (800 mg/kg per day) for 7 weeks. The control group fed a normal chow diet and the obese groups received oral administrations of the same volume of double-distilled water. The body weight and food intake of the mice belonging to these three groups were assessed once per week. The fat mass was detected in the last week of the experiment using the Minispec LF90 Body Composition Analyzer (Bruker). For the oral glucose tolerance test (OGTT), glucose (2 g/kg) was administered orally to the HFD mice after fasting for 6 h to measure their glucose tolerance, and the blood glucose levels at different time points starting immediately after the initial injection of glucose were detected using a glucometer (Accu-Check Performa, Roche Diagnostics, Basel, Switzerland). The area under the curve (AUC) of the glucose level over time was calculated to evaluate the glucose tolerance ability of the mice. Serum was collected for detection of the total cholesterol (TC), triglycerides (TG) and insulin levels using commercial kits according to the manufacturers' introductions. Feces, liver and adipose tissue was collected and stored at -80 °C.

### Metabolic rate and temperature measurements

The mouse metabolism was assessed using the Comprehensive Lab Animal Monitoring System (CLAMS, Columbus Instruments) following the manufacturer's instructions. The HFD-induced obese mice aged 12 weeks were placed in individual cages and acclimated to the monitoring system for 24 h. Their metabolic rate was assessed by continuously measuring their oxygen consumption (VO_2_), carbon dioxide production (VCO_2_) and heat production over the next 24 h. The voluntary activity resulting from the X-axis beam breaks was monitored every 30 min. The skin temperature surrounding BAT was recorded with an infrared camera and analyzed with the FLIR Research software package (FLIR Research IR Max 3.4; FLIR; West Malling).

### Histology and immunofluorescence

Mouse tissues and beige cells were fixed with 4% (wt./vol) paraformaldehyde in PBS, and paraffin-embedded sections were stained with hematoxylin and eosin for the general morphological observation. Immunofluorescence staining was performed according to the standard protocol using the following antibodies and dilutions: UCP-1 (R&D, mouse, 1:200), PRDM-16 (Abcam, rabbit, 1:200), and BODIPY 493/503 (Thermo Fisher Scientific,1:1000). The samples were first permeabilized with 0.3% Triton X-100, blocked with 5% goat serum solution, and incubated with the primary antibody in PBST containing 1% BSA overnight in a humidified chamber at 4 °C. The samples were then washed, incubated with an appropriate secondary antibody conjugated with Alexa Fluor 488/405/561 (1:1000 dilution; Invitrogen, USA) for immunofluorescence staining and counterstained with 4',6-diamidino-2-phenyl-indole (DAPI) for the staining of nuclei. The immunofluorescent images were visualized and captured using a Carl Zeiss LSM 780 system at 200× magnification.

### RNA extraction and quantitative real-time PCR

Total RNA was isolated by using RNAiso Plus Reagent (Takara, Japan), and the concentration was measured based on the absorbance ratio of 260 nm/280 nm. Subsequently, 1 µg of total RNA was reverse-transcribed into complementary DNA with a first-strand synthesis kit (Takara, Japan). For each sample, PCR was performed in triplicate with SYBR Green reagents (Takara, Japan) and the LC480 platform (Roche, USA). The expression levels of the target genes were normalized to that of the internal control (β-actin). The primer sequences are listed in Supplementary Table S1.

### Protein extraction and Western blotting analysis

Proteins were extracted with radioimmunoprecipitation assay (RIPA) lysis buffer and centrifuged at 12000 rpm and 4 °C for 10 min. The protein lysates were separated by SDS-PAGE, subsequently transferred to PVDF membranes and blocked for 2 h at room temperature. The polyvinylidene difluoride membranes were incubated with the specific primary antibodies of interest listed in Table S2 and then with the appropriate secondary antibodies (1:1000) for 2 h at room temperature. The blot was visually detected with an enhanced chemiluminescence substrate (GE Healthcare, Germany) and a Bio-Rad GelDoc imaging system. The immunoblot analysis was performed using ImageJ software, and the original scans of the immunoblots are included in Supplementary Figure S10.

### DNA extraction and 16S rRNA gene amplicon sequencing

Fecal bacterial DNA was extracted using a QIAamp DNA Stool Mini kit (QIAGEN, Germany) according to the manufacturer's instructions. The DNA concentration was detected using a 7415 Nano spectrophotometer, and the 260/280 and 260/230 ratios were measured to assess and quantify the purity of the DNA samples. The DNA samples were stored at -20 °C before use. The amplicon library was constructed *via* the amplification of the V3-V4 hypervariable regions of the 16S rRNA gene sequences using the primers (319F/806R) 26. The PCR amplification program was performed with DNA polymerase and a thermocycler using the following steps: initial denaturation at 98 °C for 2 min, 30 cycles of 98 °C for 15 s, 58 °C for 15 s, 72 °C for 15 s and a final extension at 72 °C for 3 min 26. The qualities of the PCR products were assessed using an Agilent Bioanalyzer 2100 system (Agilent, CA, USA), and the amplicon pool of PCR products was prepared with AMPure XP beads. Subsequently, the pooled PCR products were sequenced by BGI Co., Ltd., using the Illumina MiSeq sequencing system 27.

### 16S rRNA sequencing analysis

The sequencing data were subjected to quality check and read filtering using QIIME (http://qiime.org/) to obtain high-quality clean tags. Sets of sequences with a threshold identify of 97% were defined as operational taxonomic units (OTUs) using USEARCH software (v7.0.1090) 28. Representative OTU sequences were assigned using the RDP Naïve Bayesian Classifier v.2.2. The α-diversity indices (observed species, Chao 1, abundance-based coverage estimator, Simpson and Shannon) were calculated with Mothur (v1.31.2). The data analyses and the visualization of differential microbiota communities, including different OTU numbers (Venn diagram), OTU composition comparisons (principal component analysis (PCA)) and classification of the community compositions at various levels, i.e., phylum, class, order, family, genus, and species, were conducted and visualized using R (v3.1.1) software. The statistical significance of the differential microbial communities between various taxonomic groups of samples was determined using Metastats (http://metastats.cbcb.umd.edu/) and R (v3.1.1) software 29.

### Fecal microbiota transplantation

Fecal transplantations were performed according to a previous study 30; briefly, stools collected from PNS-treated HFD mice (n = 6) were pooled and resuspended in sterile PBS solution (0.1 g/mL) 31. The stool solution was vortexed and settled by gravity for 30 min. The supernatant was collected and used as the fecal transplant material (FTM). The number of viable bacteria in the supernatant was calculated using the colony-forming unit (CFU) method, and 200 μL of supernatant with approximately 9.76×10^6^ bacterial cells was administered orally to the HFD-fed recipient mice for 6 weeks 32. The body weight and food intake of the mice were assessed once per week. Six weeks after transplantation, the metabolic rate and fat mass were monitored. Adipose tissue was collected for the detection of protein expression by immunoblotting, immunohistochemistry and immunofluorescence staining. In the antibiotic treatment experiment, 5-week-old DIO mice were treated with norfloxacin and ampicillin at 1 g/L in the drinking water for 14 days 33. These mice were divided into six groups: HFD Model (water), HFD+A (antibiotics in drinking water), HFD+A+PNS (HFD+A+0.8 g/kg/day PNS) and HFD+A+FTM. The body weight was measured each week, and the fat mass was detected during the final week. Fecal samples were collected for bacterial DNA extraction and detection, and the quantitative polymerase chain reaction (qPCR) method was used to evaluate the differences in the abundances of *A. muciniphila* and *P. distasonis* among the HFD, HFD+A, HFD+A+PNS and HFD+A+FTM groups.

### Cell culture, differentiation and RNA interference

C3H101/2 cells were cultured with DMEM (Invitrogen), supplemented with 10% (v/v) fetal bovine serum (FBS, Invitrogen) at 37 °C in a 5% CO_2_ incubator. For the silencing of the AMPK α or STAT 3 genes, C3H101/2 cells (4 days after the induction of beige differentiation) were transfected with a mouse siRNA targeting STAT 3 (SC-29494) or AMPKα1/2 (sc-45313) or control siRNA (sc-37007) purchased from Santa Cruz Biotechnology (Reston, VA, USA) according to the manufacturer's instructions 34. On the day of transfection, 1.5 μL of Lipofectamine 300 (Invitrogen) and 60 nM siRNA were added separately into 100 μL of Opti-MEM medium, and the mixtures were incubated for 5 min. The two solutions were then mixed, and the resulting mixture was incubated for an additional 15 min and added to the culture wells in complete DMEM. After 12 h of incubation, the growth medium was added, and the samples were incubated for another 2-3 days 35. Adipocyte differentiation was confirmed by BODIPY 493/503 staining, which demonstrated the visual appearance of lipid droplets in green color. The browning of adipocytes was verified by q-PCR analyses of beige-specific protein. The knockdown of protein expression was confirmed by immunoblot analyses.

### Statistical analysis

The data are expressed as the means ± standard deviations from at least three individual experiments. For multiple comparisons, one-way ANOVA was performed using the analysis software Prism 8, and comparisons between two groups were assessed by a *t*-test. Tukey's test is recommended for post hoc testing. Significant differences are shown based on the *p* value and a *p* value less than 0.05 was considered to indicate statistical significance.

## Results

### PNS reduced adiposity in DIO mice but not in mice with induced obesity and impaired leptin signaling

**Figure 1:**
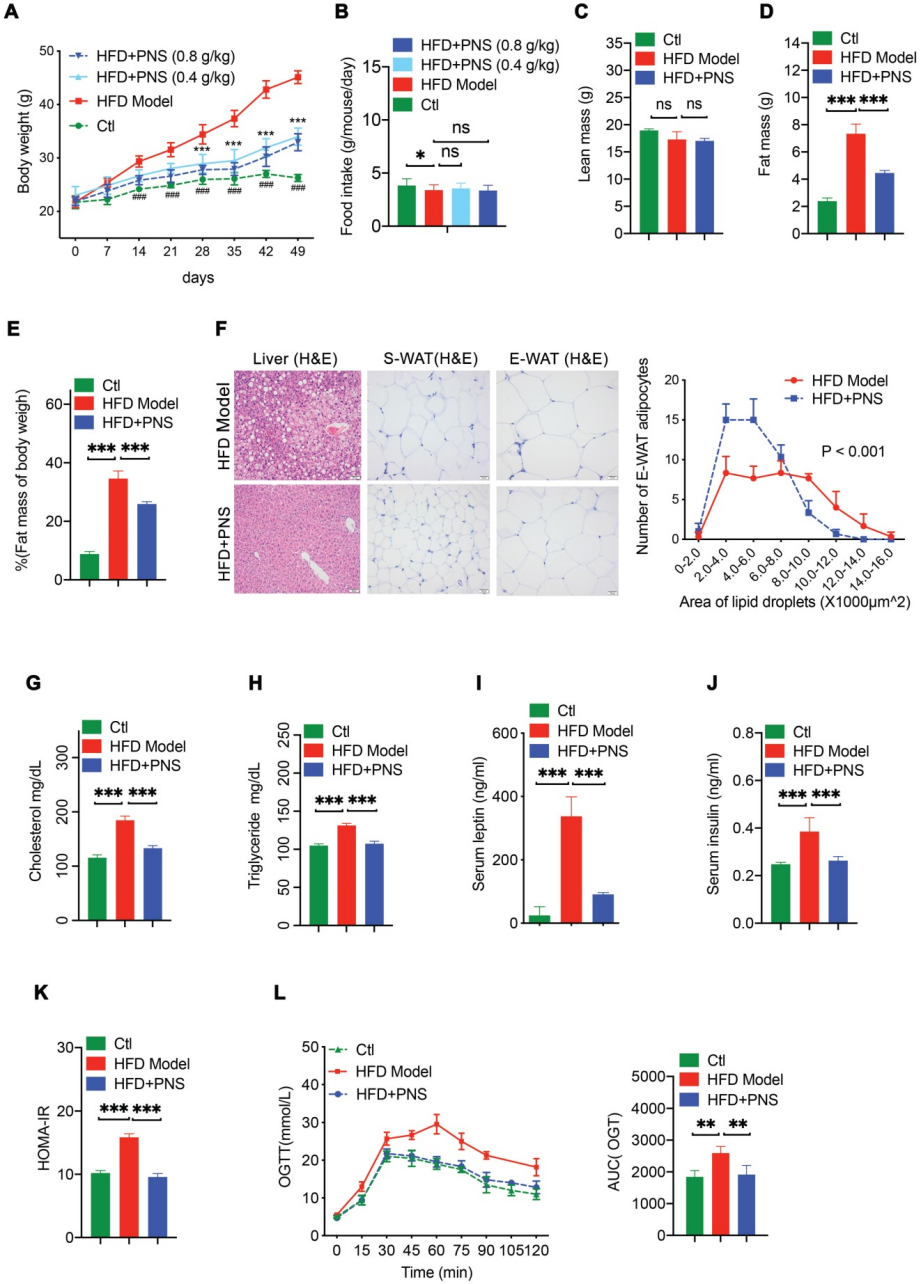
** PNS-treatment induced metabolic alterations in mice with HFD-induced obesity**. (A) Body weight of CTL and HFD mice treated with vehicle or PNS (400 or 800 mg/kg). (B) Food intake of CTL and HFD mice treated with vehicle or PNS (400 or 800 mg/kg). (C-E) MRI analysis of lean and fat mass changes in CTL and HFD mice treated with vehicle or PNS (800 mg/kg). (F) Representative images of H&E-stained liver, subcutaneous white adipose tissue (S-WAT) and epididymal white adipose tissue (E-WAT) of obese mice treated with vehicle or PNS (800 mg/kg) (scale bar: 50 µm) and relationship between the numbers and area of E-WAT lipid droplets. (G, H) Serum total cholesterol and triglyceride levels in mice treated with vehicle or PNS (800 mg/kg). (I) Serum leptin of CTL and HFD mice treated with vehicle or PNS (800 mg/kg). (J) Serum insulin of CTL and HFD mice treated with vehicle or PNS (800 mg/kg). (K) HOMA-IR index of CTL and HFD mice treated with vehicle or PNS (800 mg/kg). (L) Glucose tolerance test results of obese mice orally administrated 2 g of glucose per kg after 6 h of fasting and area under the curve (AUC) analysis of three different groups of obese mice. All the data are presented as the means ±SEMs from n = 6 mice per group. Significant differences among different groups are indicated with asterisks: *P < 0.05, **P < 0.01 and ***P < 0.001.

We investigated various body composition and white adipose features by comparing the response of C57BL/6J mice with HFD-induced obesity, leptin-deficient (ob/ob) mice and leptin receptor-deficient (db/db) mice to PNS treatment. DIO mice fed a HFD for 7 weeks showed apparent weight gain compared with the lean control (CTL) mice, and PNS treatment significantly reduced the weight gain of HFD-fed DIO mice (Figure 1A). In contrast, the effects of PNS on lowering the body weight was subtle in ob/ob and db/db mice with impaired leptin signaling (Figure S2A). Because leptin might activate the hypothalamus regulation of food intake 36, we measured whether PNS-treated mice consume less food. Unexpectedly, PNS treatment had minimal influence on the food intake of obese mice (Figure 1B and S2B) and the lean mass fraction of the body composition of DIO mice (Figure 1C) but induced a noticeable decrease in the fat mass and percentage in DIO mice (Figure 1D,1E). The fat composition of ob/ob and db/db mice was not changed by PNS treatment (Figure S2C). The fat mass reduction observed in PNS-treated mice was due to the suppression of white adipocyte accumulation. Because WATs located in different body positions have distinct impacts on the progression of obesity, we isolated two primary white fat pads, namely, subcutaneous white adipose tissue (S-WAT) and epididymal white adipose tissue (E-WAT), to evaluate the respective adipose alterations. The histopathological examination of S-WAT and E-WAT was performed using H&E staining (Figure 1F), and these observations showed that both the S-WAT and E-WAT of the HFD+PNS group exhibited smaller adipocyte sizes, but no remarkable change was detected in either adipose tissue from ob/ob or db/db mice (Figure S2D). Typical features of nonalcoholic fatty liver disease, including increases in the lipid content and degree of liver injury, can also be observed during the progression of obesity 37. The features were further alleviated by PNS treatment, as demonstrated by histological H&E staining of HFD-treated mice (Figure 1F). Obesity exerts detrimental effects on lipid metabolism, such as TC, TG and insulin production. The reduction in the TG, TC and insulin levels detected in the plasma of HFD-fed, ob/ob, and db/db mice indicated that PNS treatment could normalize these lipid metabolic parameters (Figure 1G-H and S2E). Compared with the results obtained with lean mice, the circulating level of leptin was elevated due to increases in adipose tissue in DIO mice, and PNS treatment decreased the serum leptin level (Figure 1I), which might be a consequence of reduced adiposity and weight loss 38. Moreover, the PNS-treated HFD mice showed improved insulin sensitivity (Figure 1J-K) and better tolerance to a glucose load, as demonstrated by an oral glucose tolerance test (Figure 1L). These results indicated that PNS improved lipid and glucose metabolism in a mouse model of obesity induced by a HFD and in ob/ob and db/db mice (Figure S2G); specifically, the anti-obesity effects of PNS on adipocyte formation and differentiation are potentially associated with leptin signaling.

### PNS-treated DIO mice exhibited increased energy expenditure *via* BAT activation

**Figure 2:**
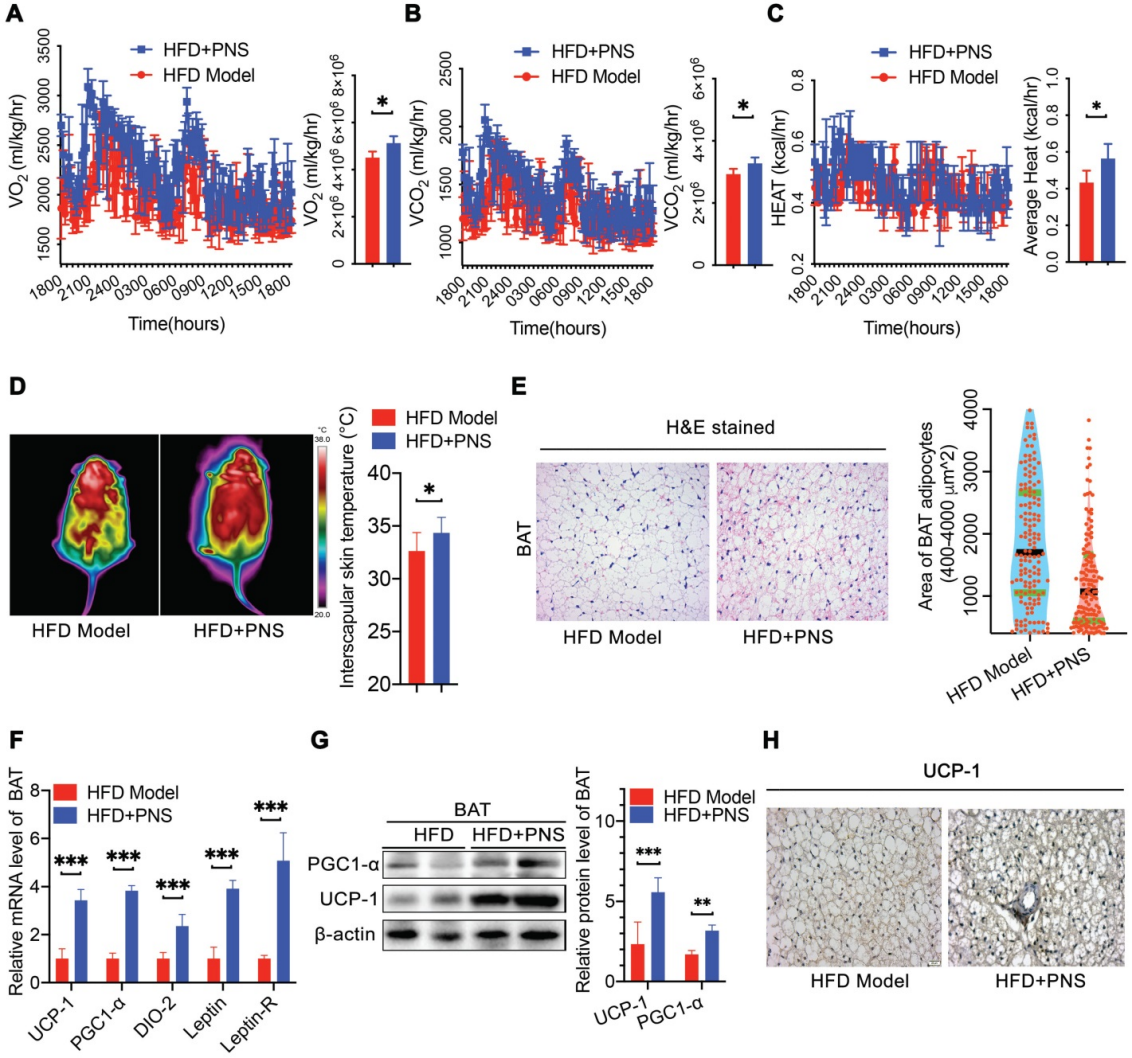
** PNS-treated mice exhibited increased energy expenditure and thermogenesis**. (A-C) Evaluation of various energy expenditure parameters and results from the average AUC analysis of oxygen consumption (VO_2_) (A), carbon dioxide (VCO_2_) (B) and heat (expressed as Kcal/h) (C) during a 12-h light/dark cycle in HFD induced obese mice treated with vehicle (HFD, n = 5) or PNS (HFD+PNS, n = 5). (D) Representative infrared thermography analysis and interscapular skin temperature of the HFD and HFD+PNS groups (n = 6 mice per group). (E) H&E-stained sections of brown adipocyte tissue of the HFD and HFD+PNS groups. (scale bar: 20 µm) and area of brown adipocytes in the HFD and HFD+PNS groups. (F) Relative mRNA expression of the BAT markers UCP-1, PGC1-α, DIO-2, leptin and leptin-R in the HFD and HFD+PNS groups. (G) UCP1 and PGC1α protein levels in BAT. β-actin was used as a loading control. (H) Staining of UCP-1 in brown adipocytes of the HFD and HFD+PNS groups (scale bar: 20 µm). The values are presented as the mean± SEMs. Significant differences compared with the vehicle controls are indicated by asterisks: *P < 0.05, **P < 0.01 and ***P < 0.001.

Obesity is caused by the loss of balance between energy intake and expenditure 39. Because we did not observe any changes in energy intake in the PNS-treated mice, we used a comprehensive laboratory animal monitoring system (CLAMS) to determine the energy expenditure. During a 12-h light/dark cycle, the HFD mice receiving PNS (HFD+PNS group) exhibited increased oxygen consumption (VO_2_) and carbon dioxide production (VCO_2_) (Figure 2A-B). The whole-body energy expenditure of the HFD+PNS mice was significantly increased (Figure 2C). To further compare the level of energy expenditure between the control and treated mice, the body temperature of the mice was tested using an infrared camera (FLIR system) for the assessment of thermogenesis. The thermal image of the mice treated with PNS reflected more areas with high body temperatures than the images of the untreated mice (Figure 2D), which suggested increased thermogenesis. However, no significant increase in body temperature was found in ob/ob and db/db mice after PNS administration (Figure S2F). BAT is responsible for energy dissipation in the form of heat, whereas the activation of BAT triggers thermogenesis, which leads to an increased body temperature 40. Consistent with the reduction in fat mass, the PNS-treated mice harbored smaller brown adipocytes associated with reduced size of lipid droplets (Figure 2E). The mRNA and protein expression of brown adipocyte-specific genes, including UCP-1, PGC-1α, and type II iodothyronine deiodinase (DIO-2), were strongly activated in HFD+PNS mice (Figure 2F). The UCP-1 protein found in the mitochondria of brown adipocytes accelerates energy expenditure and promotes thermogenesis in BAT and the browning of white adipose tissue to yield beige adipose tissue 41. The expression of UCP-1 was driven by several transcriptional components, including the coactivator PGC-1α (PGC-1α) and the enzyme DIO-2. Moreover, PGC-1α and UCP-1 have been implicated as brown adipocyte-specific genes and are highly expressed in multilocular mitochondria-rich adipocytes 42. PNS treatment robustly induced the activation of UCP-1, PGC-1α, and DIO-2, and this activation further modulates the process of thermogenesis and energy expenditure in BAT. Immunoblotting analysis indicated that UCP-1 and PGC-1α expression was significantly increased by PNS treatment (Figure 2G), and immunohistochemistry detection revealed higher expression of UCP-1 in mature brown adipocyte (Figure 2H).

### PNS-treated DIO mice showed the beige adipocyte reconstruction *via* leptin activation

**Figure 3:**
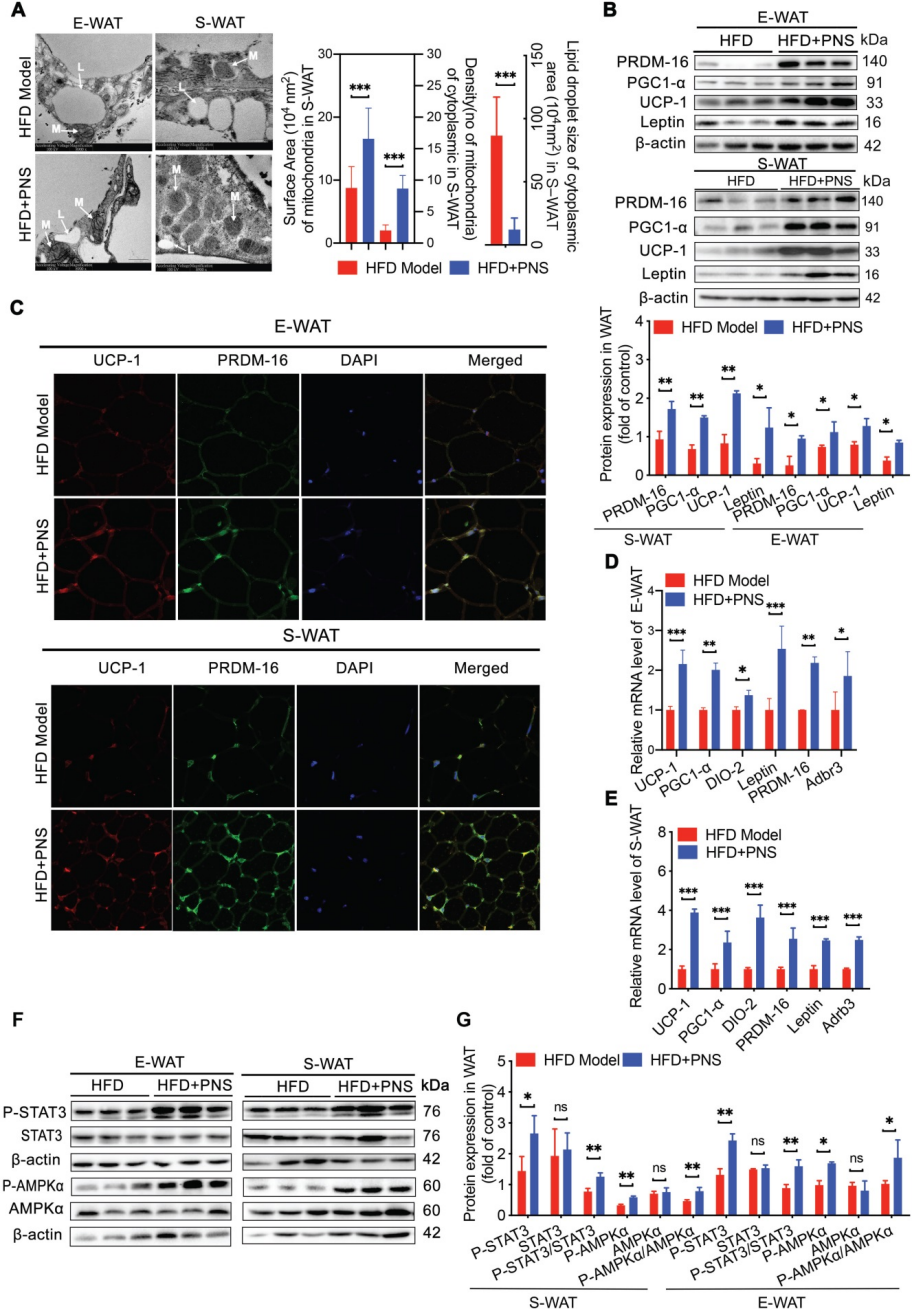
** Browning in WAT was associated with leptin activation.** (A) Representative TEM image of E-WAT and S-WAT of HFD and HFD+PNS mice (n = 6, original magnification: 8900; mitochondria, M; lipid droplet, L) and TEM image analysis of the mitochondrial number/area and the lipid droplet size in the cytoplasmic area in the S-WAT. (B) Western blot analyses of PRDM-16, PGC1-α, UCP-1 and leptin protein levels in E-WAT and S-WAT. β-actin was used as a loading control. (C) Representative fluorescence images showing the expression of UCP-1 (red) and PRDM-16 protein (green) in E-WAT and S-WAT of HFD and HFD+PNS mice. Scale bar: 20 µm. (D-E) Relative mRNA expression of the BAT markers UCP-1, PGC1-α, PRDM-16, DIO-2, leptin and leptin-R in E-WAT (D) and S-WAT (E) of HFD and HFD+PNS mice (n = 6). (F-G) Western blot analyses of the STAT3 and AMPKα protein levels and phosphorylation of STAT3 and AMPKα in E-WAT and S-WAT of HFD+PNS and HFD mice. The values are presented as the means± SEMs. Significant differences compared with the vehicle controls are indicated by asterisks: *P < 0.05, **P < 0.01 and ***P < 0.001.

White adipocytes most frequently display a single lipid droplet and a relatively low mitochondrial mass 43. A reduced white fat mass is correlated with a smaller adipocyte size, and the browning process is associated with the mitochondrial biogenesis. S-WAT and E-WAT from PNS-treated mice showed an increase in the intracellular mitochondrial numbers and a significant decrease in the size of lipid droplets, as demonstrated by transmission electronic microscopy (TEM) analysis (Figure 3A, scale bar = 500 nm), which indicated that PNS treatment could improve the energy homeostasis of white adipocytes in S-WAT and E-WAT. Moreover, the browning phenomenon in white adipocytes was accompanied by an increase in the expression of thermogenic genes or proteins. PRD1-BF1-RIZ1 homologous domain containing 16 (PRDM-16) in myoblasts appeared to promote the differentiation of adipocyte precursors to brown fat cells 44, and β3-adrenergic receptors (Adrb3) induce brown remodeling of white adipocytes. PRDM-16 contributed to the development and function of beige cells, which were involved in the browning of WAT in the PNS group (Figure 3B). Similarly, PNS treatment also increased the protein expression of UCP-1 and PGC1-α in white adipocytes (Figure 3B). A confocal microscopy analysis confirmed the increases in UCP-1 and PRDM-16 protein expression in E-WAT and S-WAT of PNS-treated DIO mice (Figure 3C). Moreover, both the S-WAT and E-WAT of the PNS group showed excellent induction of UCP-1, PRDM-16, Adrb3, PGC-1α, DIO-2 and leptin at the mRNA level (Figure 3D-E). Because the anti-adipogenesis effect of PNS in mice with deficient leptin signaling was not significant, we further investigated whether the molecular conversion of white adipocytes to beige cells induced by PNS treatment was derived from adipocyte changes caused by leptin signaling. The expression of leptin at the gene and protein levels was significantly stimulated in WAT by PNS treatment, as shown in Figure 3B, 3D and 3E. Previous studies illustrated that leptin induced signal transducer and activator of transcription 3 (STAT3) signaling further triggered adipocyte browning in mice 45, 46. Moreover, our immunoblotting analysis showed that PNS administration stimulated the phosphorylation of AMP-activated protein kinase-α (AMPKα) protein and the phosphorylation of STAT3 in primary adipocytes (Figure 3F-3G). In summary, our findings showed that the browning of WAT in the HFD+PNS group was promoted by the leptin-AMPKα/ STAT3 signaling pathway.

### PNS-treated DIO mice harbored a distinct gut microbiota profile that contributes to reduced obesity

**Figure 4:**
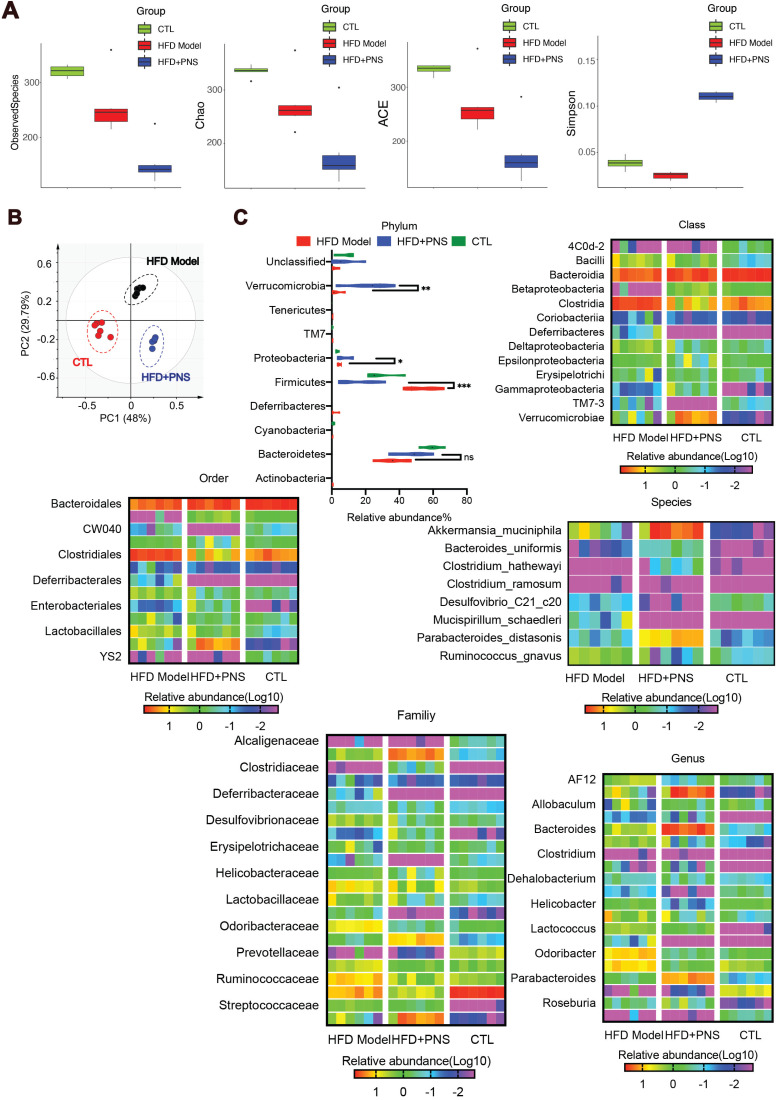
**PNS altered the microbiota composition of HFD mice.** (A) Box-plot of the α diversity of the CTL, HFD model and HFD+PNS groups. (B) PCA score plot analysis based on the relative abundance of OTUs. A different color indicates each group of mice (n = 6), and each spot represents one mouse. (C) Analysis of the significance of the bacterial taxonomic profiling at the phylum level and the heat map showing the different relative abundances of the bacterial community at the class, order, family, genus and species levels in HFD+PNS and HFD mice.

Obesity has been characterized by specific alterations in the abundance and function of the gut microbiota 47. We investigated the effects of PNS on the community structure of the gut microbiota in diet-induced obese mice by 16S rRNA gene-based profiling of the fecal microbiota. The decreased total number of OTUs at 97% sequence similarity in the PNS-treated group might indicate a lower degree of diversity compared with the HFD-fed obese model (Figure S3A). We found that the OTU richness and evenness represented in the rank abundance curve were visually shifted after PNS treatment (Figure S3B). To assess the α diversity of the gut microbiota, five indices, including observed species, Chao 1(reflecting the species richness), abundance-based coverage estimator (ACE, similar to Chao 1) and Simpson indices (estimating the species diversity), were calculated using Mothur (v1.31.2). As shown in Figure 4A, the boxplot visually indicated a statistically significant difference in α-diversity among the CTL, HFD+PNS and HFD groups. Compared with the CTL group, the HFD group presented a decrease in α-diversity in the gut microbiota, in accordance with previous studies that showed that a decrease in α-diversity is associated with obesity 48, but these findings do not demonstrate a causal relationship 49. The reduced values of the observed species, Chao and ACE index demonstrated that the apparent lower richness of microbiota community and the high Simpson index showed significantly increased species diversity in the DIO mice after PNS treatment. A PCA revealed separation among the CTL, HFD and HFD+PNS individuals based on the first two principal component (PC) scores, which accounted for 48% and 29.79% of the total variations (Figure 4B). This result indicated that the overall gut microbial communities of the DIO mice were strongly shaped by PNS. The β diversity analysis supported the above-described result and indicated a significant difference in complexity between the HFD and HFD+PNS mice, as shown by the weighted UniFrac-based heat map of the β diversity (Figure S3C). The clustering of the species composition showed that the low similarity in species composition among samples from two different groups, particularly the HFD and HFD+PNS groups (Figure S3D). Taxonomy-based comparisons of the gut microbiota were performed to compare the overall community structure of the gut microbiota of the different groups. Histograms of the distributions of the taxonomic composition at the phylum, class, order, family, genus and species levels are shown in Figure 4C-H 50. Among all the bacterial groups, *Firmicutes* was the most predominant phylum, contributing relative abundances of 27.21, 53.66 and 15.78% to the gut microbiota composition of the CTL, HFD and HFD+PNS mice, respectively, followed by *Bacteroidetes* (68.20, 53.89 and 37.14%). The phylum analysis revealed that the ratio of *Firmicutes* to *Bacteroidetes* in HFD-fed mice was reversed by PNS treatment (Figure 4C). The statistical analysis showed significant increases in the relative abundances of *Verrucomicrobia* and *Proteobacteria* after PNS treatment. At the genus level, PNS treatment significantly triggered differences in species abundance, as reflected in the heat map of the species clustering analysis (Figure 4G). A heat map of individual species confirmed that PNS increased the relative abundances of *Akkermansia muciniphila* and *Parabacteroides distasonis* and reduced the level of *Ruminococcus gnavus* (Figure 4H). The predominant species, *Akkermansia* and* Parabacteroides distasonis*, exhibited firm evolutionary relationships with some other species, as shown in Figure S3E, which indicated that PNS might affect the gut bacterial ecosystem. In conclusion, we found that the diversity and richness of the gut microbial community were shifted after PNS treatment and that the alterations in the species composition were associated with increased abundances of *A.muciniphila* and *Parabacteroides distasonis*.

Based on the results from the 16S rRNA gene sequencing analysis of ob/ob mice, the obesity-related microbiota composition in DIO and ob/ob mice were compared to assess the impact of PNS treatment on the gut microbiota community in obese mice. A PCA of the weighted UniFrac distances analysis of the gut microbiota (Figure S4A) showed that the ob/ob+PNS and ob/ob mice were clustered into two separate groups, which indicated that these mice harbor a different gut microbiota community. Thus, PNS plays a significant role in shaping the microbiome, and our experiments showed that PNS could induce shifts in the gut microbiota in both DIO and ob/ob mice. Consistent with the results obtained for DIO mice, PNS intervention moderately reduced the α diversity of the gut microbiota in ob/ob mice (Figure S4B). At the phylum level, the gut microbiota in both DIO mice and ob/ob mice exhibited a high ratio of *Firmicutes* to *Bacteroidetes*, which reflects the state of obesity-related metabolic disorders 51. As shown in Figure S4C, the oral administration of PNS to ob/ob mice resulted in a markedly lower *Firmicutes-*to-*Bacteroidetes* ratio and an increased abundance of *Proteobacteria*, which is consistent with the gut microbiota of PNS-treated DIO mice. The PNS-induced gut microbiomes of ob/ob mice and HFD mice were distinguished mainly by the relative abundances of *Tenericutes* and *Verrucomicrobia*, respectively. A hierarchical clustering analysis of the genus-level bacterial community (Figure S4D) revealed that PNS offset significant alterations in the community structure of the gut microbiome of ob/ob mice, and these effects included substantial decreases in the relative abundances of *Anaeroplasma, Oscillibacter* and *Ruminiclostridium* and an increase in the level of *Parabacteroides*. Moreover, the specific genus-level bacterial distribution in HFD+PNS mice significantly differed from those in ob/ob and ob/ob+PNS mice (Figure S4E). The species-level analysis revealed that the ob/ob+PNS mice showed a significant increase in the abundance of *Parabacteroides distasonis* (Figure S4F) but a relatively lower abundance of *Parabacteroides distasonis* (8.74% in HFD+PNS mice vs. 0.625% in ob/ob+PNS mice), and no apparent alteration in the proportion of *Akkermansia muciniphila* was detected. Unlike the results observed in DIO mice, the PNS-induced modifications in the gut microbiota could not reverse the development of obesity in leptin-deficient mice, which suggested that defective leptin signaling is responsible for the negative effects of the PNS-induced modulations in the gut microbiota on adipose tissue.

### PNS exerted modulating effects on the gut microbiota associated with leptin activation

**Figure 5:**
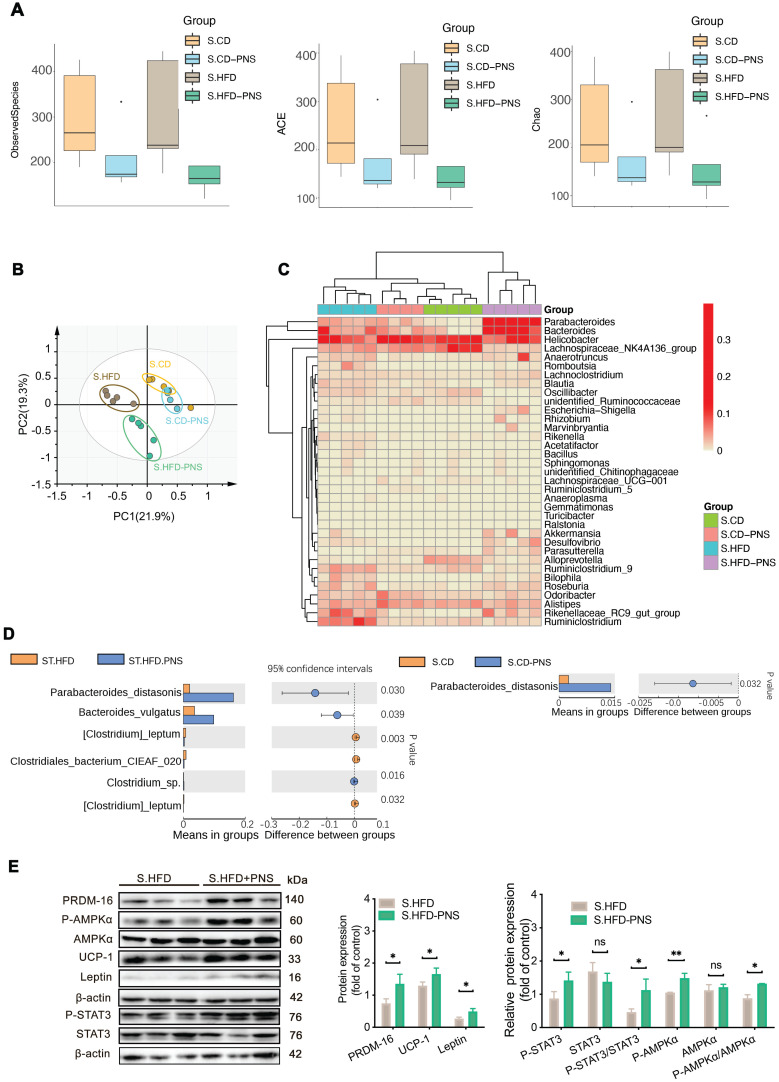
** The gut microbiota alterations associated with leptin activation contributed to the effects of PNSs.** (A) Box plot of the α diversity of the S.CD (n = 5), S.CD-PNS (n = 4), S.HFD (n = 5) and S.HFD-PNS (n = 5) groups. (B) PCA plots of the β diversity of the S.CD, S.CD-PNS, S.HFD and S.HFD-PNS groups. (C) Hierarchical clustering of the bacterial phyla in the S.CD, S.CD-PNS, S.HFD and S.HFD-PNS groups. (D) Different analyses at the bacterial species level between the untreated and PNS-treated groups. (E) Western blot analyses of the PRDM-16, leptin, STAT3, and AMPKα protein levels and the phosphorylated STAT3 and AMPKα protein levels in S-WAT of the S.HFD and S.HFD-PNS groups. β-actin was used as a loading control.

To confirm whether the specific gut microbiota alterations that are directly modulated by PNS, as well as evaluate whether PNS-induced gut microbiota is associated with leptin activation, we performed 16S rRNA gene sequencing analysis of gut microbiota in short-term PNS-treated mice fed either a chow diet (S.CD) or a high-fat diet (S.HFD) before body weight change. A similar trend of α diversity decrease was detected in S.CD-PNS and S.HFD-PNS group, indicating that short-term PNS supplementation changed the complexity of bacterial diversity in mice fed either a chow diet or HFD (Figure 5A). A PCA of the β diversity (Figure 5B) and a hierarchical clustering analysis of the bacterial phyla of the S.HFD and S.CD groups indicated that the S.CD and S.HFD mice harbored significantly different bacterial communities. In addition, short-term PNS treatment resulted in a structural rearrangement of the overall gut microbial communities in S.CD and S.HFD mice, and the rearrangement in S.CD-PNS mice showed some overlap with that in the S.CD mice (Figure 5B-C). The bacterial phyla *Parabacteroides* and *Bacteroides* were clearly increased in the S.HFD-PNS group compared with the S.HFD group, in accordance with the results obtained with long-term PNS treatment in HFD mice (Figure 5C). We found that short-term PNS treatment led to significant alterations in the abundances of bacterial species, as shown in Figure 5D, and a higher level of *Parabacteroides distasonis* was clearly observed in the S.CD-PNS and S.HFD-PNS groups (16.2% in the S.HFD-PNS group vs. 1.4% in the S.CD-PNS group), which indicated that PNSs could exert a similar modulating effect on the gut microbial ecology of mice with different diets. In particular, the DIO mice had a distal gut microbiota with an increased capacity to reduce host adiposity. Moreover, our immunoblotting analysis showed that short-term PNS administration to HFD-fed mice increased the protein expression levels of leptin, PRDM-16 and UCP-1 and stimulated the phosphorylation of AMPKα and STAT3 in S-WAT (Figure 5E), which indicated that increases in beige adipocyte reconstruction are induced by activation of the leptin-AMPKα/STAT3 signaling pathway. Thus, the beneficial effects of PNS on the microbiome are expected to contribute to brown remodeling *via* leptin regulation in DIO mice.

### Preparation and identification of fecal transfer microbiota

**Figure 6:**
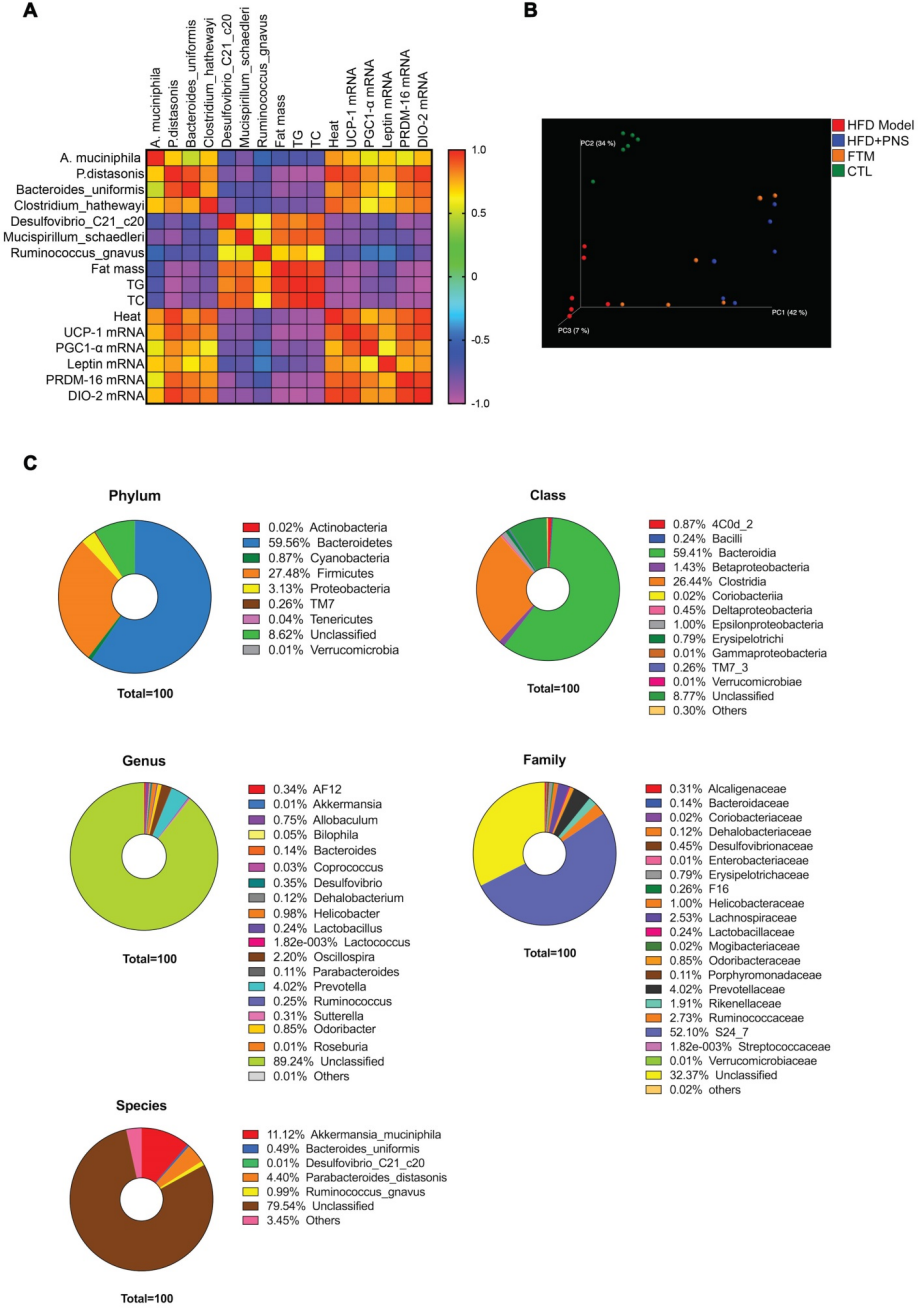
** Identification of PNS microbiota** (A) Heat map of the Spearman r correlations between the bacterial genera and the metabolic parameters measured in HFD mice treated with vehicle or PNS (n = 6/group). (B) The 3D PCoA plot of the β diversity based on the weighted UniFrac distances among the HFD, HFD+PNS, CTL and FMT groups. (C) Bacterial taxonomic profiling of bacteria from FTM at the phylum, class, genus, species and family level.

To address whether the bacterial taxa modulated by PNS were correlated with adiposity function, we constructed a correlation map by analyzing various adiposity-related metabolic parameters and determined whether these specific metabolic parameters are correlated with the specific bacterial taxa 52. As shown in Figure 6A, the correlation matrix is highlighted by different colors, where a column intersecting a row indicates a correlation. The high correlation score indicated that the alterations in the bacterial taxa were strongly correlated with adipose activities; thus, the PNS-induced modifications in the gut microbiota might trigger leptin-induced adipocyte functions. To confirm the correlation between PNS-modulated gut microbiota and leptin-induced beige cell action, fecal microbiota transplantation (FMT) was performed using fecal microbiota extracted from PNS-treated DIO mice, and this material is referred to as FTM. First, the chemicals in the fecal samples collected from PNS-treated DIO mice were analyzed *via* HPLC analysis, and a small amount of residual PNS was detected in the fecal samples (Figure S1B). Thus, the fecal samples used for FMT were free of PNS. The metabolites in the FTM detected by gas chromatography-mass spectrometry (GC-MS) analysis were mainly identified as lactic acid, glycerol, uracil, palmitic acid, oleic acid, stearic acid, palmitoylglycerol and 1-monopalmitin (Figure S5). Compared with the HFD model, we observed a distinct clustering of fecal metabolite features in PNS-treated mice (Figure S6A). The modulating effects of PNS on the gut microbiota involved changes in bacterial fermentation metabolites; for example, higher levels of fatty acids (e.g., oleic acid, phosphonic acid, and lactic acid) were observed in PNS-treated mice, as shown in Figure S6B. Bacteria-derived fatty acids are able to intervene in adipocyte function 53. The oral administration of lactate at a concentration of 357 μmol can induce UCP-1 and mitochondrial activity in the BAT of mice 54, but the fecal lactate concentration in mice is approximately 0.03 μmol/mg 55. Thus, the intermediate metabolites in the FTM diluted from fecal samples were considered to be at lower levels than those that could induce thermogenic programs. Furthermore, we performed a 16S rRNA gene sequencing analysis of the gut microbiota in the FTM, and the 3D PCoA plot of the weighted UniFrac-based β diversity (Figure 6B) indicated that the bacteria community diversity of the FTM was similar to that of the HFD+PNS group. The microbiome analysis of the FTM showed the relative abundances of bacterial taxa at the phylum, class, order, family, genus and species levels (Figure 6C). At the species level, *Akkermansia muciniphila* and *Parabaceroides distasonis,* which constitute 11.2% and 4.4% of the gut microbiota community, respectively, have been suggested as the predominant bacteria in the FTM.

### The transplant of gut microbiota triggered beige adipocyte reconstruction *via* leptin activation

**Figure 7:**
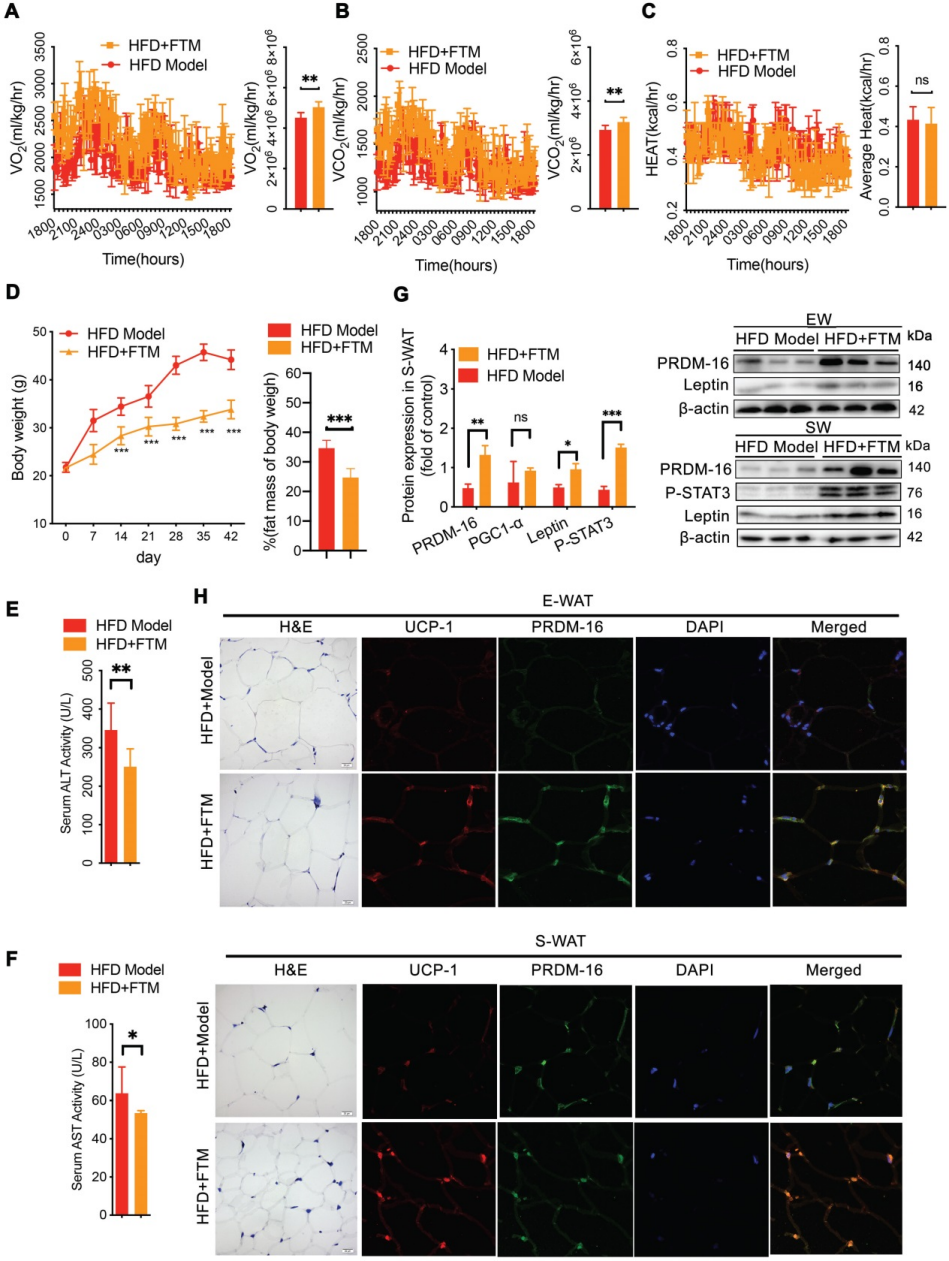
** Transfer of FTM promoted leptin-induced browning remodeling in white adipocytes**. (A-C) Energy expenditure and average AUC analysis of oxygen consumption (VO_2_), carbon dioxide (VCO_2_) and heat (expressed as Kcal/h) during a 12-h light/dark cycle in HFD+FTM and HFD mice. (D) Body weight and body composition of HFD mice treated with vehicle or FTM (n = 6). (E-F) Serum ALT and AST of HFD mice treated with vehicle or FTM. (G) Relative protein expression of PRDM-16, leptin and phosphorylated STAT3 in HFD-fed mice treated with vehicle or FTM. (H) Representative fluorescence images showing the expression of UCP-1 (red) and PRDM-16 protein (green) in E-WAT and S-WAT of HFD mice treated with vehicle or FTM.

To confirm whether the PNS-induced microbiota contributes to white adipocyte browning, the identified microbiota (FTM) was transplanted into HFD mice. The HFD mice that received the FTM (HFD+FTM group) exhibited increased oxygen consumption (VO_2_) and carbon dioxide production (VCO_2_) (Figure 7A-B). The whole-body energy expenditure of the HFD+PNS mice was significantly increased (Figure 7A-C). The oral administration of the FTM reduced the body weight and fat mass (Figure 7D) and improved the serum levels of AST and ALT in DIO mice (Figure 7E-F). Similarly, transplantation of the FTM increased the protein expression of leptin and PRDM-16 in both S-WAT and E-WAT (Figure 7G), and the leptin/STAT3 pathway was involved in these effects. The browning of white adipose tissue in the HFD+FTM group was promoted by the increased levels of UCP-1 and PRDM16 expression, as observed by confocal microscopy analysis (Figure 7H).

### The antibiotics-induced depletion of the gut microbiota eliminated FMT-mediated adipose remodeling

**Figure 8:**
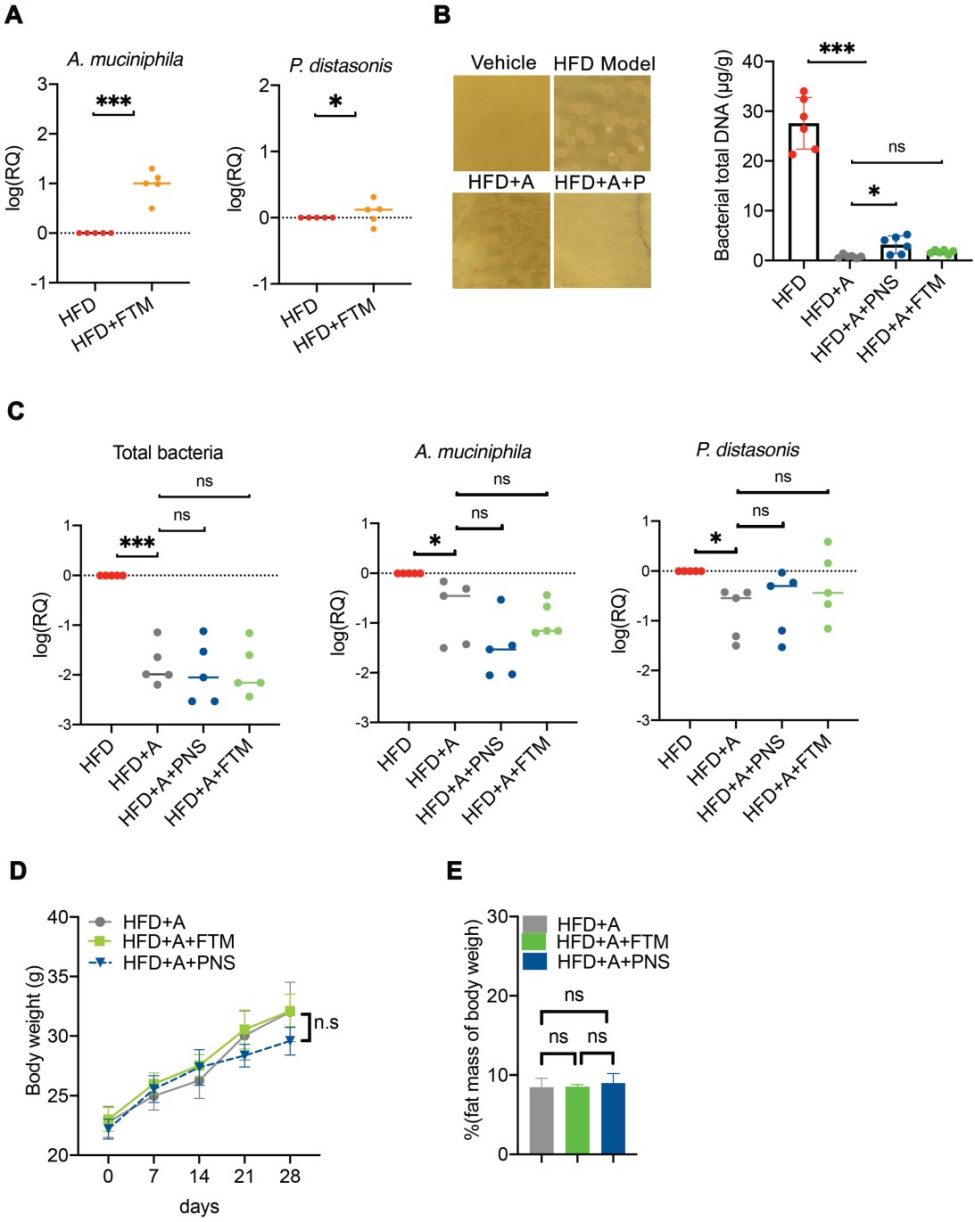
** Antibiotic-induced depletion of gut microbiota eliminated the FTM mediated adipose remodeling.** (A) Quantitative polymerase chain reaction (qPCR) validation of the differences in the abundances of *A. muciniphila* and *P. distasonis*. Each dot represents an individual mouse (n = 5). (B) The fecal microbiota were plated on LB agar plates for the assessment of bacteria growth. Bacterial growth was suppressed in HFD-fed mice by the administration of norfloxacin and ampicillin, as shown by the DNA concentrations of total bacteria in the HFD, HFD+A, HFD+A+FTM, and HFD+PNS groups. (C) qPCR validation of the differences in the abundances of total bacteria, *A. muciniphila* and *P. distasonis*. Each dot represents an individual mouse. (D) Body weight and fat mass of pseudo germ-free HFD-fed mice treated with vehicle or PNS.

To further confirm whether the PNS-induced modulation of the gut microbiota was indispensable for the anti-obesity effect, a combination of two broad-spectrum antibiotic mixtures was adopted to suppress or remove the majority of the gut microbiota in DIO mice (Figure S7). Transplantation of the PNS-modulated microbiota increased the abundance of *Akkermansia muciniphila* and *Parabaceroides distasonis* (Figure 8A) in the HFD+FTM group. Treatment with ampicillin and norfloxacin at 1 g/L in drinking water resulted in the strongest inhibition of anaerobic and aerobic bacteria in obese mice 33, 56, and the reduced abundance of intestinal bacteria in the antibiotic-treated mice (HFD+A) was confirmed by detecting the amount of bacterial DNA (Figure 8B). The antibiotic-treated HFD-fed mice were then administered PNS (HFD+A+PNS) or FTM (HFD+A+FTM) for 4 weeks. Detection of the universal 16S rRNA of total bacteria by q-PCR revealed no significant changes in the total bacteria level or the growth of *A. muciniphila* and *P. distasonis* among the HFD+A+PNS and HFD+A+FTM groups (Figure 8C). Moreover, PNS and the FTM were unable to cause significant reductions in body weight and fat mass in the antibiotic-treated HFD-fed mice (Figure 8D). These findings were consistent with the above-mentioned data, which indicated that the anti-obesity effects of PNS were mediated by gut microbiota and that the altered gut microbiota could induce WAT browning *via* the leptin signaling pathway.

### The cell-autonomous effect of leptin but not PNS was involved in the gut microbiota-induced reconstruction of beige adipocytes *via* AMPKα/STAT3 activation

**Figure 9:**
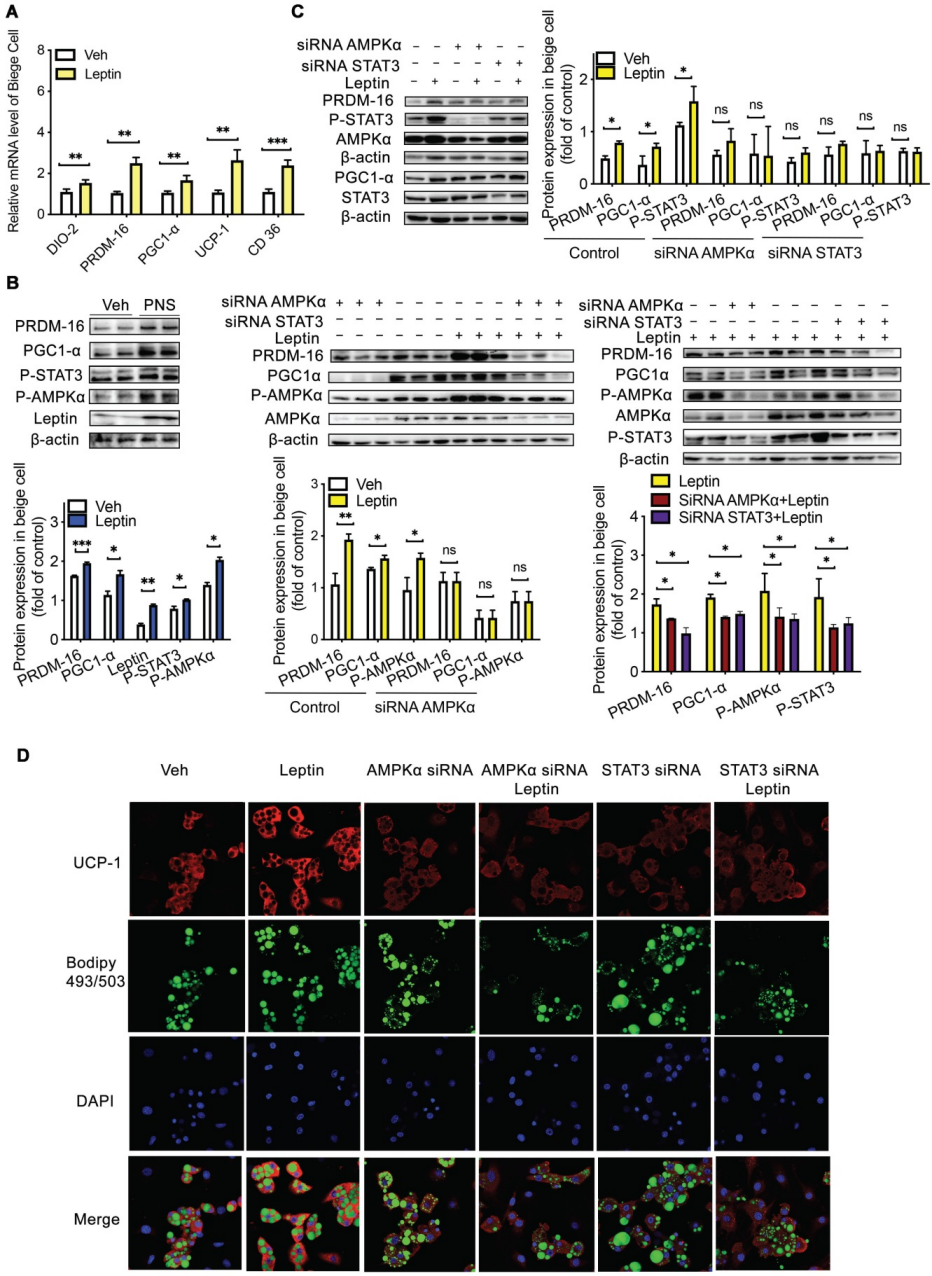
**Leptin activation was dependent on the AMPKα/STAT3 pathway.** (A) Relative mRNA expression of the BAT markers UCP-1, PGC1α, PRDM-16, DIO-2, and CD36 in differentiated C3H10T1/2 cells treated with vehicle, leptin or PNS. (B) Western blot analyses of PRDM-16, PGC1α, leptin, and phosphorylated STAT3 and AMPKα protein levels in differentiated C3H10T1/2 cells. β-actin was used as a loading control. (C) The efficiency of the siRNA-mediated depletion of AMPKα and STAT3 in beige cells treated with leptin was determined by immunoblotting. (D) Representative fluorescence images showing the expression of UCP-1 (red) and the status of lipid droplets stained with BODIPY 493/503 (green) in the siRNA-treated cells after induction. Scale bar: 20 µm.

First, we assessed whether PNS and leptin exert cell-autonomous effects on the browning process 57 using mesenchymal precursor cells (C3H10T1/2) 58. Interestingly, we found that after 24 h of incubation with 50 μg/mL PNS, these saponins could enter C3H10T1/2 cells, as shown in Figure S8A. PNS and leptin (10 nM) positively influenced the differentiation of C3H10T1/2 into adipocytes *via* increases in the expression of UCP-1, DIO-2, PRDM-16, PGC1α, and CD36 (Figure 9A), and PNS increased the phosphorylation of STAT3 and AMPKα, which are proteins downstream of leptin (Figure 9B). These results suggested that both leptin and PNS might directly influence the browning process in a cell-autonomous manner. However, most PNS with very low drug permeability are poorly absorbed into the human body and are rarely distributed in humans to achieve their efficacies. Thus, our *in vitro* observations might not actually reflect the *in vivo* situation. A LC-MS analysis showed that Rg1, Rb1 and NR1 did not enter adipose tissue (Figure S8B-C), which indicated that trace levels of PNS did not enter adipose tissue to exert their effects *in vivo* directly. Thus, even if PNS exhibit robust effects in cell culture, the therapeutically relevant actions of PNS in animal models might contribute to modulations in the gut microbiota that lead to systemic changes in metabolism and subsequent induction of browning. Furthermore, to examine whether the leptin signaling pathway induces the expression of browning markers through the activation of AMPKα and STAT3, we transiently transfected C3H10T1/2 beige cells with siRNA specific to AMPKα and STAT3, respectively. The knockdown of AMPKα and STAT3 neutralized the effect of leptin (10 nM) on PRDM-16 and PGC1α activation (Figure 9C). As shown by confocal microscopy (Figure 9D), adipocyte differentiation is commonly visualized with the lipid-specific stain BODIPY (493/503), and browning is verified by UCP-1-positive beige cells. The increased expression of UCP-1 in differentiated C3H10T1/2 cells induced by leptin was also reversed after the depletion of AMPKα or STAT3. Moreover, AMPKα depletion blocked the phosphorylation of STAT3, but STAT3 depletion did not influence AMPKα, which further suggested that the regulatory effects of the leptin-STAT3 pathway were mediated by AMPKα activation. Taken together, the results indicate that leptin stimulates the AMPKα/STAT3 pathway to exert cell-autonomous effects on the differentiation of C3H10T1/2 cells into beige adipocytes, and the direct influence of leptin on browning *via* the AMPKα/STAT3 signaling pathway support our *in vivo* findings that signaling through the leptin-AMPK/STAT3 pathway induced by PNS-modulated gut microbiota is involved in beige adipocyte reconstruction.

## Discussion

The gut microbiota has been extensively investigated due to its involvement in the pharmacological activities of PNS 21, 23, 24, 59, 60. Our findings showed that the anti-obesity effects of PNS were mediated by the gut microbiota in DIO mice, in accordance with previous clinical studies, which showed the anti-obesity effects of ginsenosides are dependent on the gut microbiota of obese women patients 22, 61. The gut microbiota has been proven to be an important endogenous factor modulating BAT activity and the browning of WAT 62, and gut microbiota changes in obese patients are positively linked to the expression of PRDM16 in S-WAT 62. The induction of browning through the transplantation of beneficial gut microbiota has been proved to be an effective therapeutic strategy for treating obesity 11, 63, 64 in experimental animal studies 65. At present, the effects of FMT as a therapy for obesity and related metabolic disorders have been assessed in clinical studies 66. However, FMT products are not commercially available because safety assessments cannot satisfy the requirements of novel food regulations 67. In our study, the PNS-induced modulation of the gut microbiota increased BAT thermogenesis, induced beige adipocyte reconstruction, and thus promoted energy expenditure in DIO mice. Our findings provide a promising therapeutic intervention for the treatment of obesity that targets gut microbiota by activating WAT browning.

In HFD-fed mice, PNS supplementation markedly shifted the HFD-induced gut microbial ecology prior to changes in body weight, which indicated that PNS could directly influence the gut microbial community, and these effects particularly included increases in the level of *Parabacteroides distasonis* within a short time. PNS interfered with the complexity of the bacterial diversity and increased the abundance of *Parabacteroides distasonis* in both CD and HFD mice, which indicated that PNS exert a similar modulating effect on the gut microbial ecology of mice with different diets. As a result, the distal gut microbiota of DIO might exhibit a significantly increased capacity to reduce adiposity. Therefore, the diet might not be the determining factor in the PNS-controlled changes in the gut microbiota. Moreover, we confirmed that PNS could reduce the body weight in both the CD and HFD groups and prevented the DIO mice from gaining weight when fed an HFD (Figure S9A). However, the PNS-induced alterations in the gut microbiota could not reverse obesity in leptin-deficient mice, which showed that defective leptin signaling is considered responsible for the negative effects of the PNS-mediated modulation of the gut microbiota on adiposity.

PNS-induced thermogenesis and beige cell recruitment were predominantly observed in wild-type mice but gene-deficient mice with impaired leptin pathway signaling, which indicated that the anti-obesity effects of PNS were associated with leptin activation. The leptin-dependent effect appears to be a result of sympathetic gut function-dependent interactions between the PNS and the gut microbiota. The alerted α-diversity observed after PNS intervention supported the previous finding that the modulation of the gut microbiota to exert anti-obesity effects is not positively correlated with the microbiota diversity 68. When considering therapeutic interventions, restrictive diets and antibiotics promote reductions in the α-diversity of the gut microbial community 68, but the gut microbiota diversity is increased after bariatric surgery (Roux-en-Y gastric bypass) 69; therefore, we hypothesized that alterations in the gut microbiota diversity might be associated with the type of therapeutic intervention other than weight loss. The shape of the intestinal barrier structure (Figure S9B) involving mucin-2 interactions could be considered a PNS-mediated protective mechanism associated with mucin-degrading bacteria (Figure S9C). A metagenomic analysis of PNS-treated DIO mice revealed that PNS increased the abundance of *Akkermansia muciniphila* and reduced the proportions of *Ruminococcus gnavus* in HFD-fed mice. As mucin-degrading bacteria, *A. muciniphila* and *Parabacteroides distasonis* have been found to be negatively correlated with diabetes, obesity and other metabolic syndromes. *A. muciniphila* reverses HFD-induced obesity by modulating the adipocyte metabolism and gut barrier function 70. *Parabacteroides distasonis* can reduce obesity and related metabolic disorders through the generation of succinate and secondary bile acids 71. Conversely, *Ruminococcus gnavus* produces an inflammatory polysaccharide in the gut 72, and the reduced abundance of *Ruminococcus gnavus* induced by PNS could improve inflammatory symptoms in obesity. Our modified microbiota transplantation intervention triggered the progression of brown remodeling *via* the leptin/STAT3 signaling pathway, and the noted decrease in fat mass after gut microbiota transplantation was abrogated by antibiotics; these findings indicated that microbiota transplantation played a vital role in leptin-induced WAT browning.

Published evidence showed that bacteria-derived long-chain fatty acids are involved in the ginsenoside-induced modulation of the gut microbiota to affect BAT activity 73. Gut microbiota-induced changes in the fatty acid composition can interfere with adipocyte activity 53; for example, bacteria-derived short-chain fatty acids including propionate can increase leptin expression *via* the G protein-coupled receptor GPR41 74. Lactate can induce UCP-1 and mitochondrial activity for adaptive thermogenesis 54, and some specific lactic acid bacteria can regulate the leptin level in adipocytes 75; Oleic acid supplementation in 3T3-L1 cells can increase the level of leptin 30. We found higher levels of fatty acids (such as oleic acid, phosphonic acid, and lactic acid) in PNS-treated DIO mice, as shown in Figure S5. This finding might suggest that the PNS-induced alterations in the gut microbiota potentially promote the generation of some bacteria-derived metabolites and subsequently trigger leptin-mediated WAT browning. However, how these alterations by the gut microbiota are involved in the activation of leptin-AMPKα/STAT3 signaling to promote beige reconstruction is worthy of further study.

Previous studies have noted that the leptin-associated brain-adipose axis plays a relevant role on BAT thermogenesis and browning 75 and on the control of food intake by the central nervous system, which results in the induction of weight loss 13. However, DIO mice are resistant to the anorectic actions of leptin due to a reduction in leptin transport across the blood-brain barrier or a deficiency in the leptin receptor-related signaling pathway in the central nervous system 76, which might explain why PNS had minimal influence on leptin-regulated food intake. Although obesity is characterized by a diminished anorectic response to exogenous leptin in the central nervous system, DIO mice still exhibit leptin actions, and leptin can even increase thermogenesis in interscapular BAT *via* a hypothalamus-independent pathway 77. In mice, leptin is able to preserve an obese status 78 and triggers the browning of white adipocytes by the Hedgehog signaling pathway 46. Leptin-JAK2-Stat3 signaling induced by FOXC2 can mediate the alleviation of adipocyte inflammation and subsequently interacts with PRDM-16 to promote beige cell formation 45. In our study, we found that PRDM-16 and PGC1α, as the primary regulators of mitochondrial biogenesis, were triggered by leptin in an AMPK/STAT3-dependent manner to promote beige adipocyte recruitment in DIO mice. The direct influence of leptin on browning *via* the AMPKα/STAT3 signaling pathway in a cell-autonomous manner supported our *in vivo* results, which showed that signaling through the leptin-AMPK/STAT3 pathway induced by the PNS-mediated modulations in the gut microbiota was involved in beige adipocyte reconstruction.

Although the anti-obesity effects of PNS mediated by gut microbiota were associated with the leptin-induced thermogenic program, the impact of PNS on the obesity-related metabolic phenotype might be correlated with another leptin-independent mechanism. For example, PNS can decrease the cholesterol levels by activating ABCG5 and ABCG8, which target LXRα to induce cholesterol secretion into the intestinal lumen 79. PNS can lessen cholesterol synthesis by decreasing hepatic HMG-CoA reductase and promote the synthesis of bile acids from cholesterol by upregulating the expression of CYP7A1 79. Moreover, PNS can improve insulin resistance and ameliorate glucose intolerance 80 through the stimulation of glucose transporter type 4 (GLUT4) and the IRS1-PI3K-AKT signaling pathway 81. PNS can also reduce inflammation in diabetic rats and attenuate high glucose-induced injury *via* the antioxidant defense system 82. Additionally, we cannot exclude the possibility that additional mechanisms are triggered by PNS-induced modulations of the gut microbiota. We found that PNS-treated ob/ob mice exhibited decreased levels of *Lachnospiraceae*, which might alleviate the development of diabetes in mice 83. The increased *Parabacteroides distasonis* can reduce hyperglycemia *via* the promotion of intestinal gluconeogenesis 71*.* These findings might support previous findings showing that PNS exert a leptin-independent metabolic effect to improve the obesity-related metabolic phenotype in obese mice 84. Overall, we concluded that the anti-obesity effects of PNS were mediated by leptin activation induced by changes in the gut microbiota, and this finding offers new insights into the microbiota-mediated regulation of beige fat and provides a novel anti-obese therapeutic strategy involving modulation of the gut microbiota.

## Supplementary Material

Supplementary figures and tables.

## Abbreviations

BAT: Brown adipose tissue
WAT: white adipose tissue
S-WAT: subcutaneous adipose tissue
E-WAT: epididymal adipose tissue
H&E: Hematoxylin and eosin
qRT-PCR: Quantitative real time polymerase chain reaction
FTM: fecal transplant material
FMT: fecal microbiota transplantation
DIO: diet induced obesity
HFD: High fat diet
PNS: Panax Notoginseng saponins
AMPK: AMP-activated protein kinase-alpha
STAT3: transcription 3
UCP-1: uncoupling protein 1
PGC1-α: Peroxisome proliferator-activated receptor gamma coactivator 1-alpha
PRDM-16: PR domain-containing 16
DIO-2: Type II iodothyronine deiodinase
Adrb3: β3-adrenergic receptors
OGTT: Oral glucose tolerance test
AUC: The area under the curve of the plot
TC): TC, total cholesterol
TG): TG, triglycerides
OTUs: operational taxonomic units
ACE: Abundance-based Coverage Estimator
PCA: Principal component analysis
GC-MS/MS: gas chromatography-tandem mass spectrometry
UHPLC: ultra-high-performance liquid chromatography
TEM: Transmission electron microscopy
