# Supplementary material for: Panax notoginseng saponins modulate the gut microbiota to promote thermogenesis and beige adipocyte reconstruction via leptin-mediated AMPKα/STAT3 signaling in diet-induced obesity
Source: Theranostics. 2020 Sep 14;10(24):11302–23. doi: 10.7150/thno.47746 (PMC7532683; doi:10.7150/thno.47746)
Supplement: Supplementary file 1 — Supplementary figures and tables. [file thnov10p11302s1.pdf]

Supplementary Materials

A

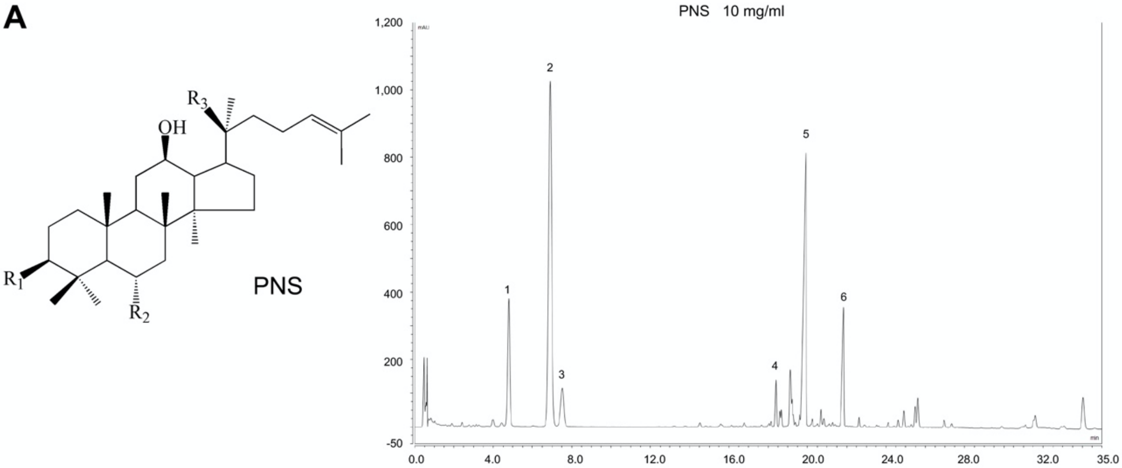

Compound	R1	R2	R3
Notoginsenoside R1(1)	-OH	-O-Glc-(2-1)-Xyl	-O-Glc
Ginsenoside Rb1(5)	-O-Glc-(2-1)-Glc	-H	-O-Glc-(6-1)-Glc
Ginsenoside Rd(6)	-O-Glc-(2-1)-Glc	-H	-O-Glc
Ginsenoside Re(3)	-OH	-O-Glc-(2-1)-Rha	-O-Glc
Ginsenoside Rf(4)	-OH	-O-Glc-(2-1)-Glc	-OH
Ginsenoside Rg1(2)	-OH	-O-Glc	-O-Glc

B

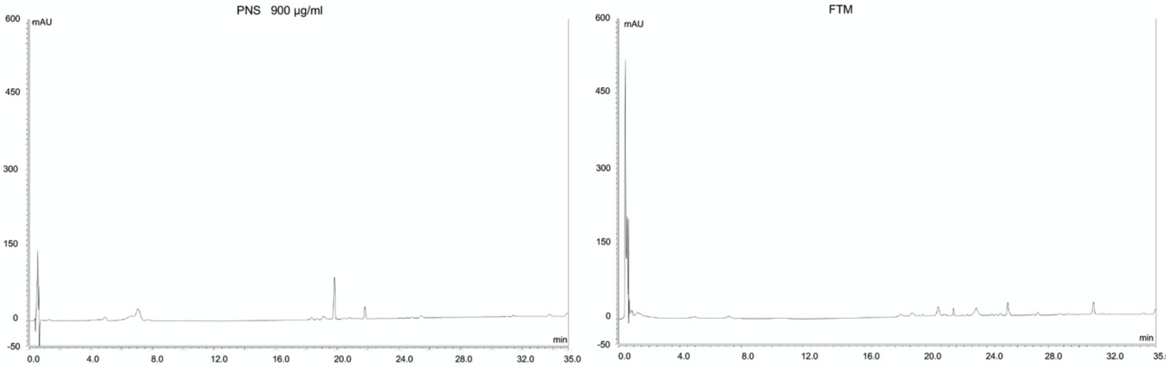

Figure S1 | UPLC-UV profiles (A) Chemical structure of compounds in PNS and (1) Notoginsenoside R1(2) Ginsenoside Rg1(3) Ginsenoside Re (4) Ginsenoside Rf (5) Ginsenoside Rb1 (6) and Ginsenoside Rd contained in PNS sample. (B) PNS sample of 900 μg/ml and FTM (fecal transplant material**)**

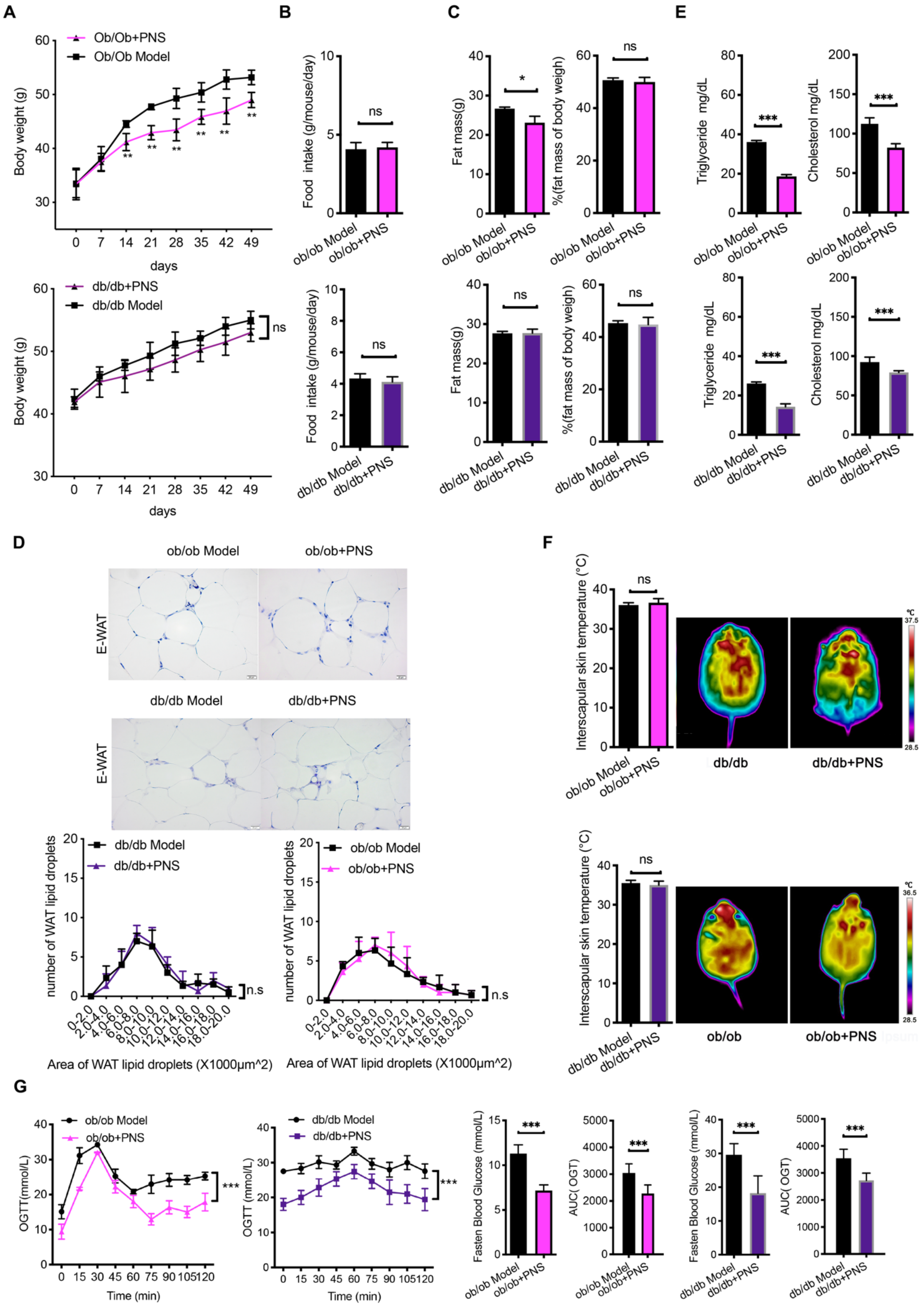
A
B
C
E
D
F
G
Ob/Ob+PNS
Ob/Ob Model
db/db+PNS
db/db Model
Body weight (g)
days
Food intake (g/mouse/day)
Fat mass(g)
%(fat mass of body weigh)
Triglyceride mg/dL
Cholesterol mg/dL
ob/ob Model
ob/ob+PNS
db/db Model
db/db+PNS
E-WAT
number of WAT lipid droplets
Area of WAT lipid droplets (X1000μm^2)
Interscapular skin temperature (°C)
db/db
db/db+PNS
ob/ob
ob/ob+PNS
OGTT(mmol/L)
OGTT (mmol/L)
Time (min)
Fasten Blood Glucose (mmol/L)
AUC(OGT)
AUC(OGT)
ns
n.s
***

Figure S2 | PNS fails to reduce fat mass in ob/ob and db/db obese mice. (A) Body weight evaluation in ob/ob and db/db mice treated with vehicle or PNS (800mg/kg). (B) Food intake of ob/ob and db/db mice treated with vehicle or PNS (800mg/kg). (C) MRI analysis of Fat mass change in ob/ob and db/db mice treated with vehicle or PNS (800mg/kg). (D) The representative images of H&E stained Subcutaneous White Adipose Tissue (S-WAT) and Epididymal White Adipose Tissue (E-WAT) of obese mice treated with vehicle or PNS (800mg/kg) (Scale bar: 20 μm.) and The relation between numbers of E-WAT lipid droplets and Area of E-WAT lipid droplets in ob/ob and db/db obese mice. (E) Serum Total Cholesterol (TC) and Triglyceride (TG) levels in ob/ob and db/db mice treated with vehicle or PNS (800mg/kg). (F) Representative infrared thermography analysis and interscapular skin temperature detection in ob/ob and db/db mice treated with vehicle or PNS groups, n = 5 mice per group. (G) Glucose tolerance test results of ob/ob and db/db mice orally administrated 2 g of glucose per kg after 6 h of fasting and area under the curve (AUC) analysis of ob/ob and db/db mice treated with vehicle or PNS. Significant differences between different groups indicated as *P < 0.05, **P < 0.01, ***P < 0.001.

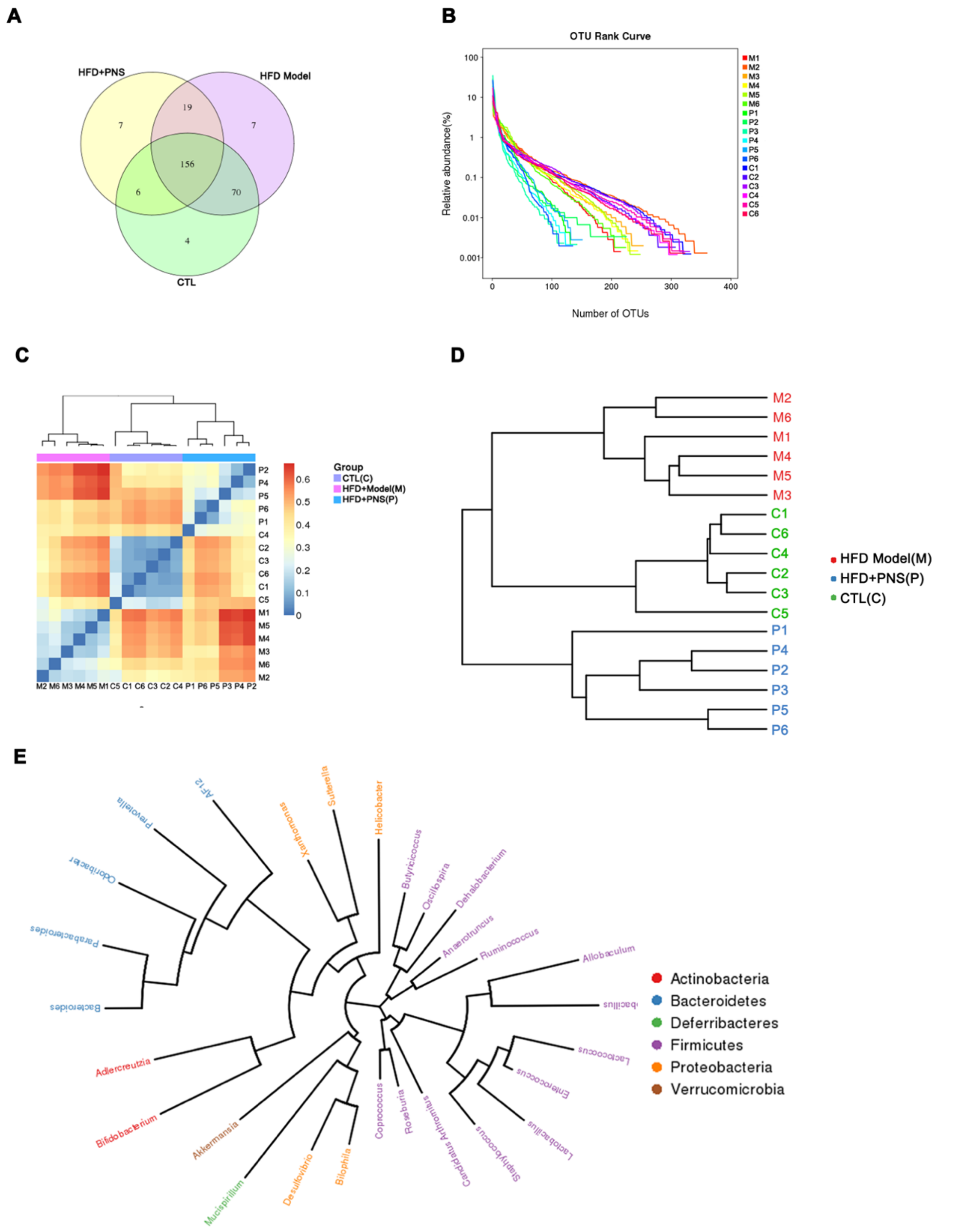

Figure S3 | Analysis of community patterns of gut microbiota from different groups (A) Shared OTU across the CTL, HFD, HFD+PNS groups (B) OTU Rank Curve of different groups (C) The heat map of the β diversity (D) Samples Clustering result (Description, weighted UniFrac). (E) A phylogenetic tree among various biological species based upon similarities and differences in their physical or genetic characteristics.

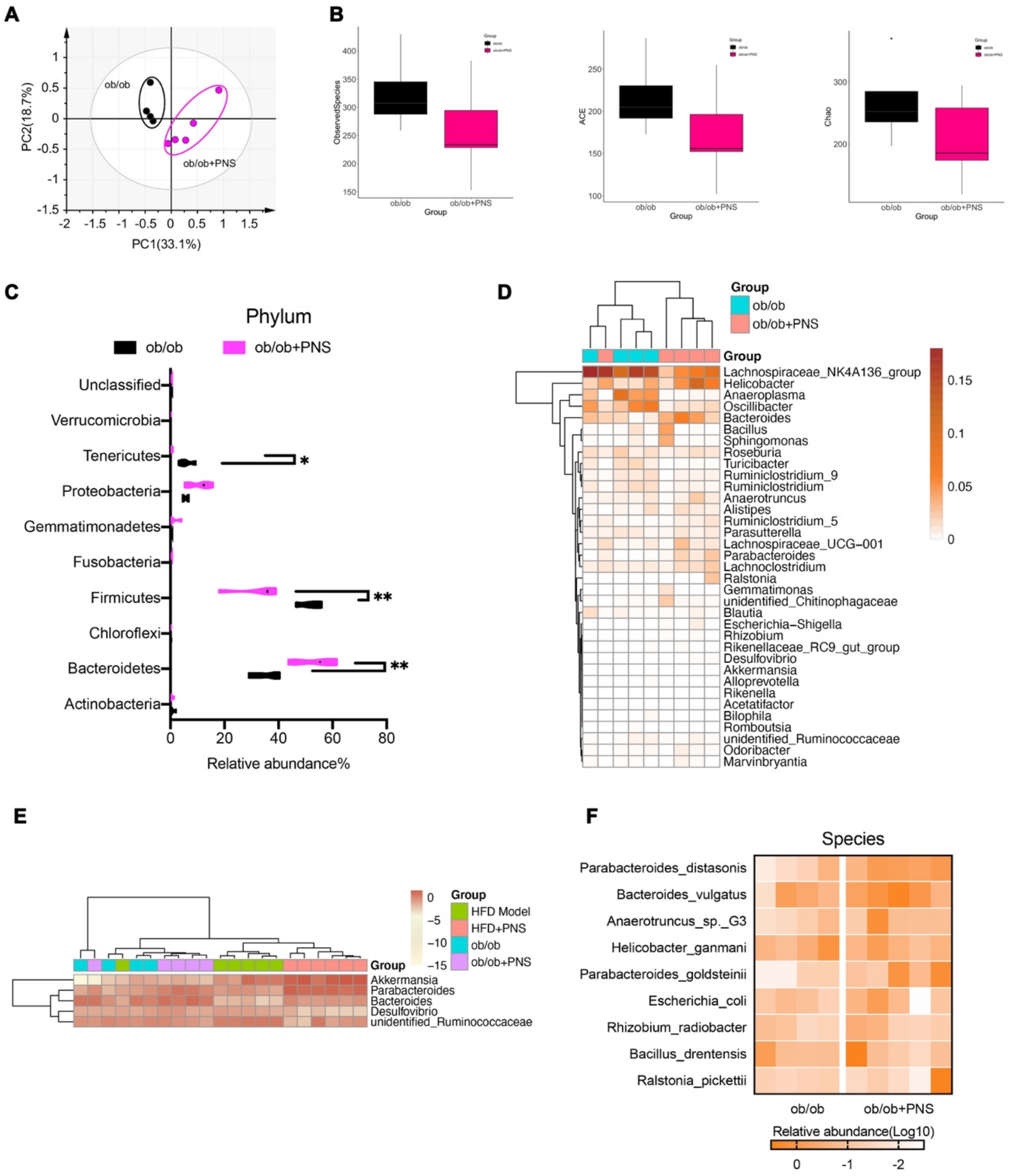

Figure S4 | Analysis of microbiota community in ob/ob mice (A) The PCA plot of the β diversity analysis in the ob/ob and ob/ob +PNS groups. (B) Box-plot of α diversity analysis of the different groups. (C) The significance analysis of bacterial taxonomic profiling at phylum level. (D) Hierarchical clustering of bacterial genus in ob/ob and ob/ob+PNS groups. (E) Hierarchical clustering of bacterial species among HFD, HFD+PNS, ob/ob and ob/ob+PNS

groups. (F) The heat map showing the different relative abundances of bacterial community at species level in the ob/ob and ob/ob +PNS groups.

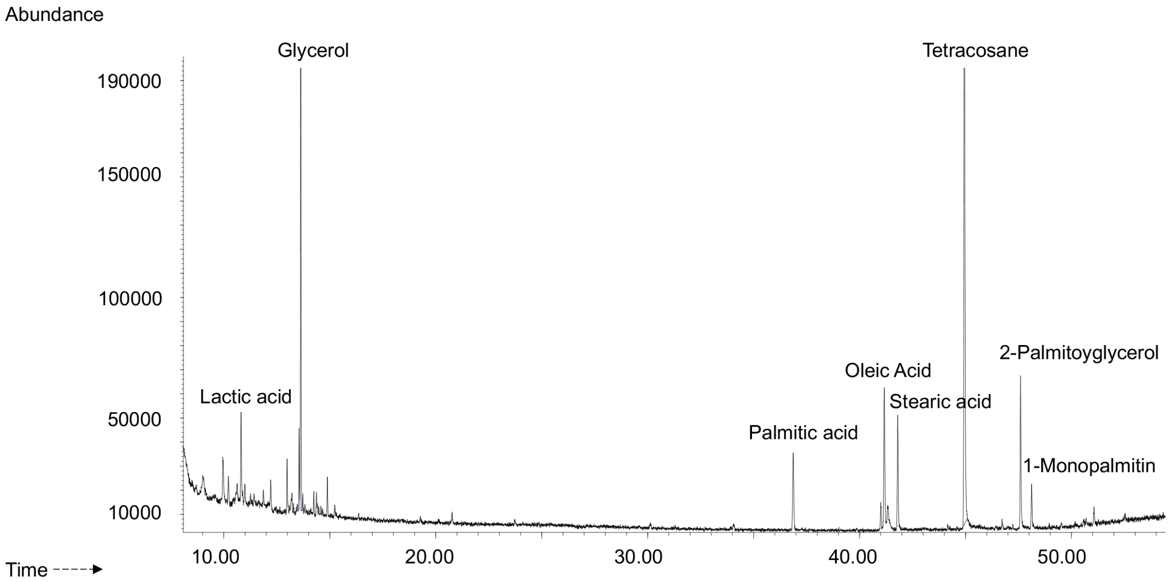

Figure S5 | GC-MS profiles of FTM (fecal transplant material)

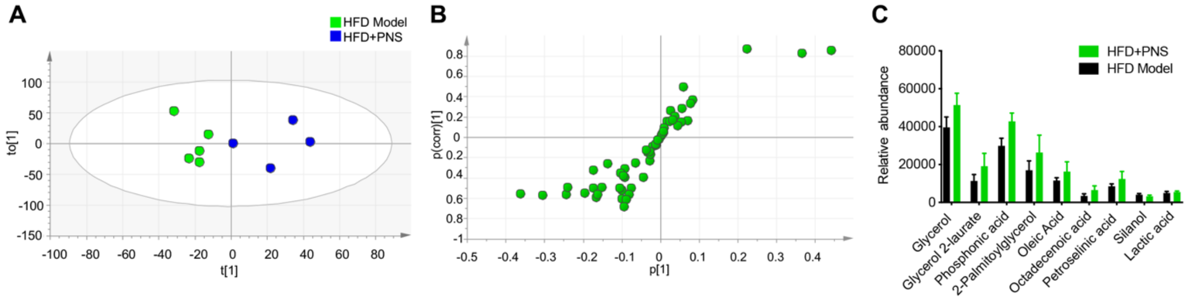

Figure S6| PNS altered fecal fatty acid metabolic profiles. (A) Orthogonal partial least-squares discriminant analysis (OPLS-DA) score plot showing group separation in fecal metabolic profile of HFD and HFD+PNS groups. (B) The OPLS-DA loading S-plot. (C) The relative abundance of fecal metabolites of HFD Model and HFD+PNS groups by GC-MS analysis.

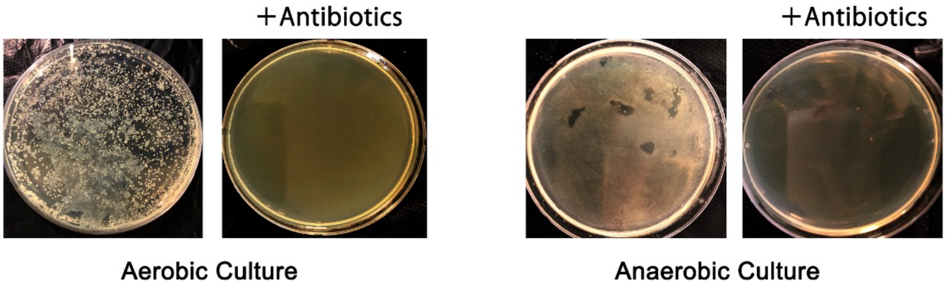

Figure S7| The plates of overnight bacterial culture of stool samples with antibiotics or not

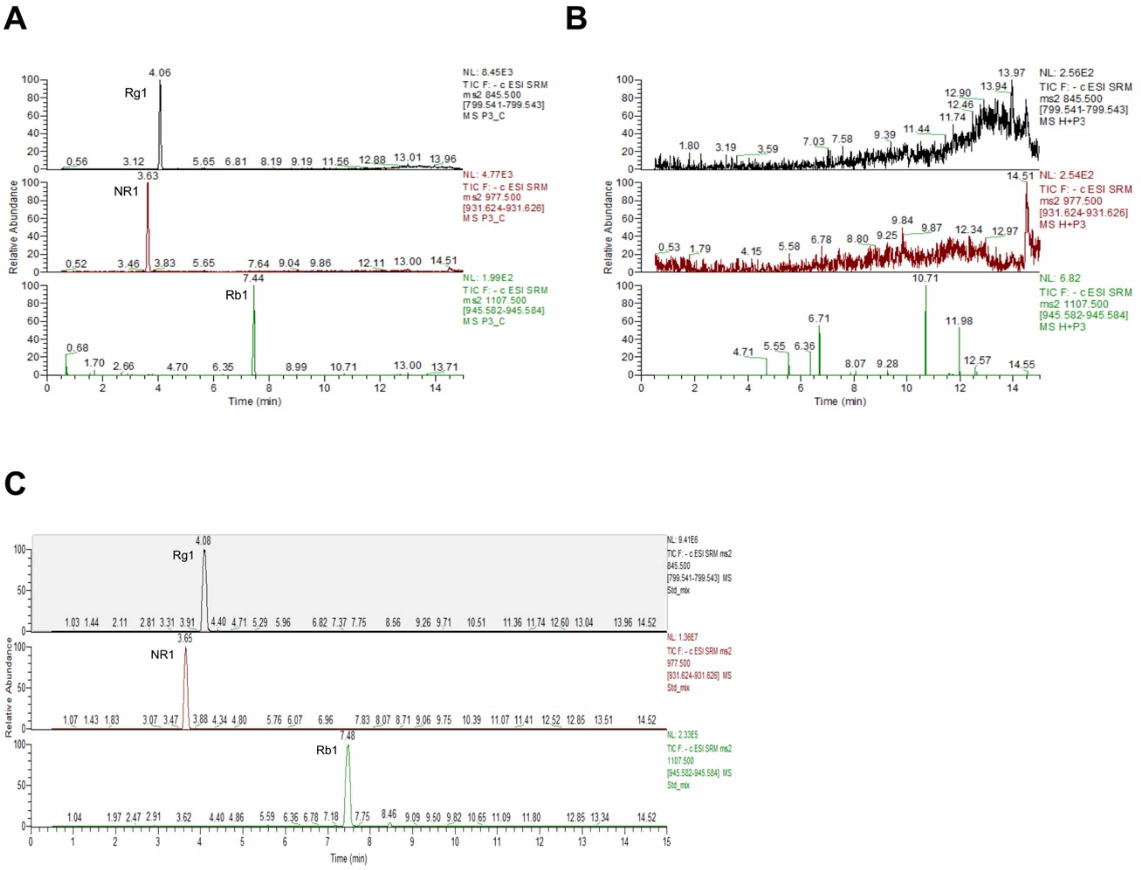

Figure S8| The detection of Rg1, Rb1 and NR1 in PNS by LC-MS analysis. (A) Rg1, Rb1 and NR1 in C3H10T1/2 cells by LC-MS analysis. (B) None of Rg1, Rb1 and NR1 in adipose of HFD+PNS group. (C) Standard chemicals of Rg1, Rb1 and NR1 detection by LC-MS analysis.

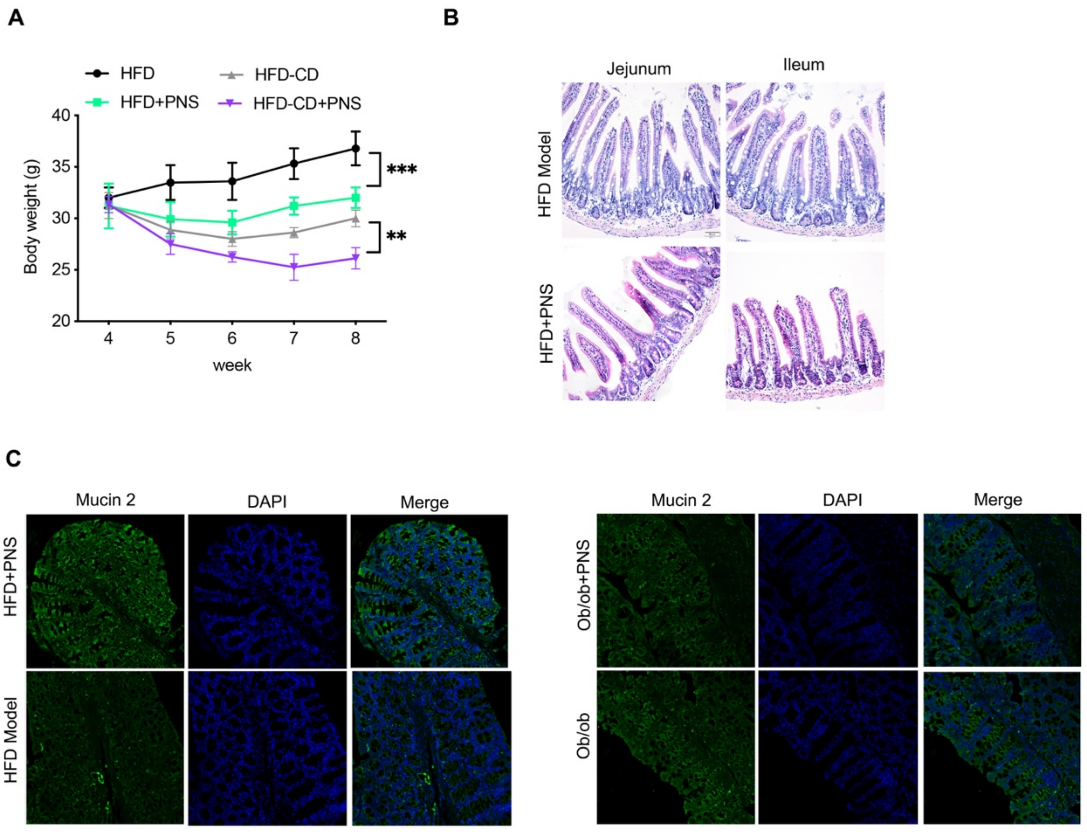

Figure S9| PNS interference was associated with intestinal barrier alteration. (A) After 4-week HFD induction, DIO mice fed HFD or CD were treated with PNS or vehicle and the body weight was assessed during 4-week treatment. (B) Representative H&E staining (Scale bar: 50 μm) of small intestine in different groups. (C) Changes in Mucin-2 expression by immunofluorescence staining in colon section of HFD and ob/ob mice with vehicle or PNS.

Fig.2G

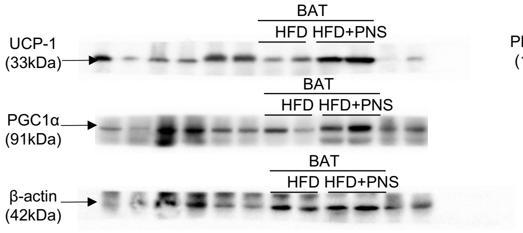

Fig.3B

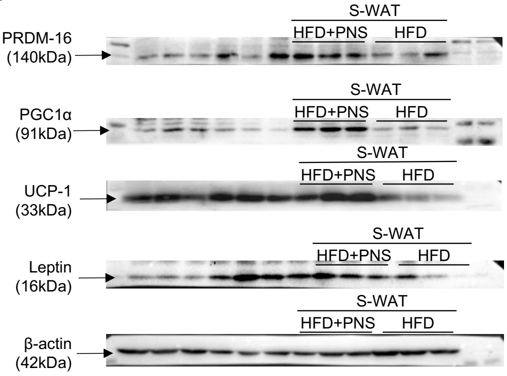

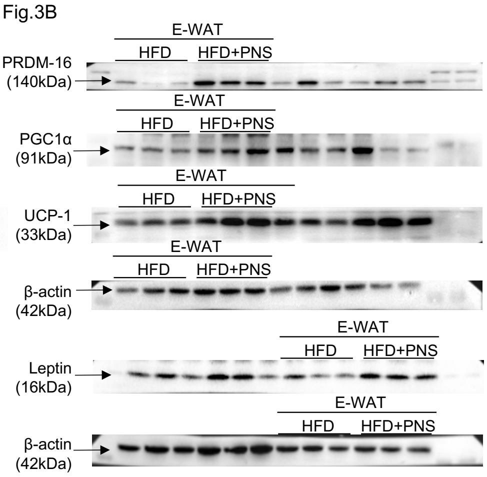

Fig.3F

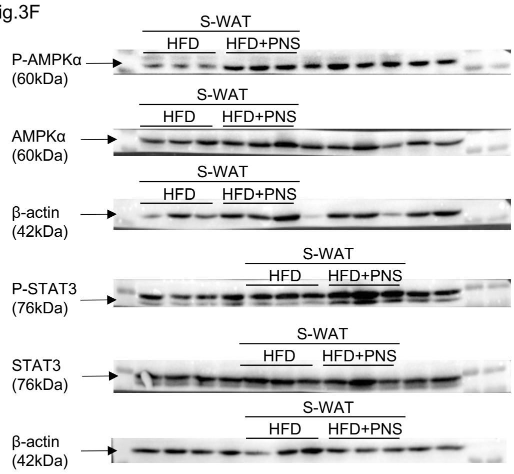

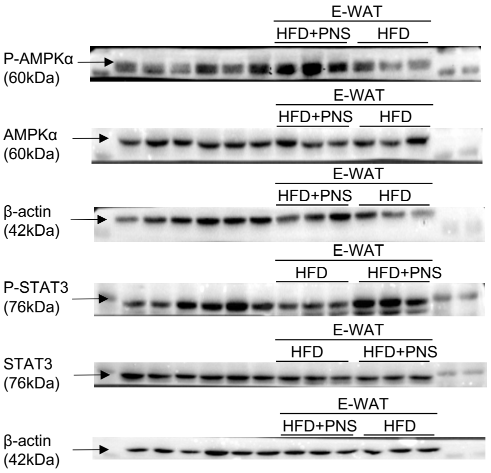

Fig.4B

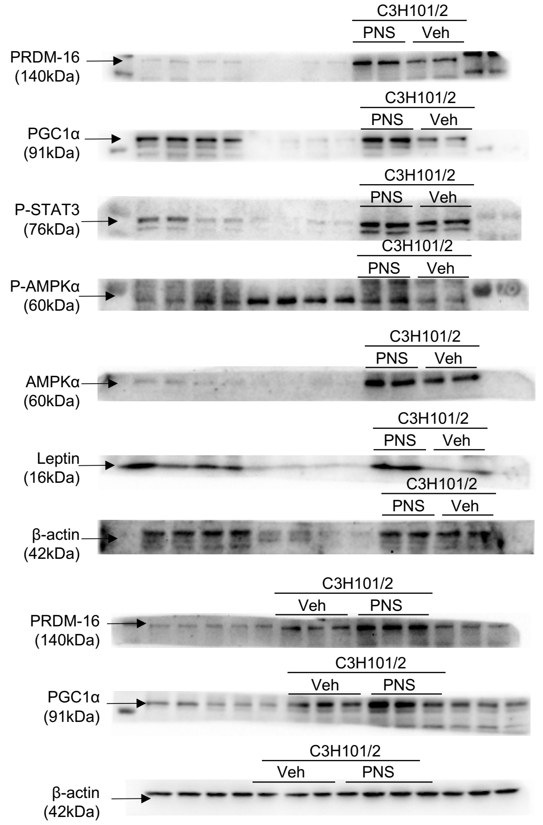

Fig.4C

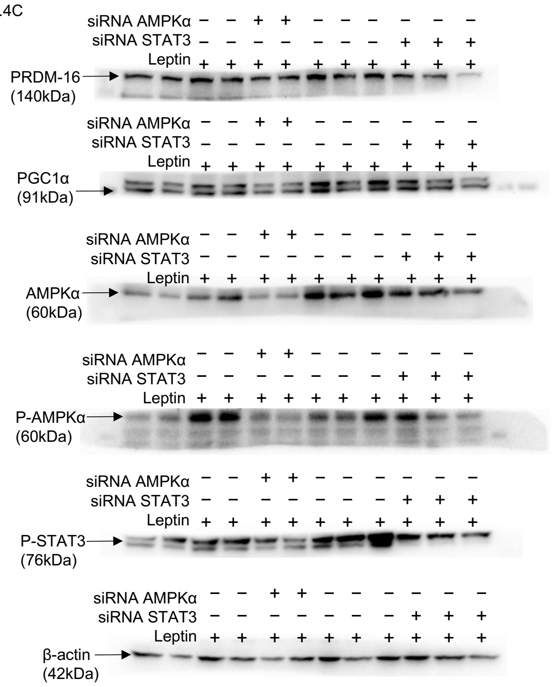

Fig.4C

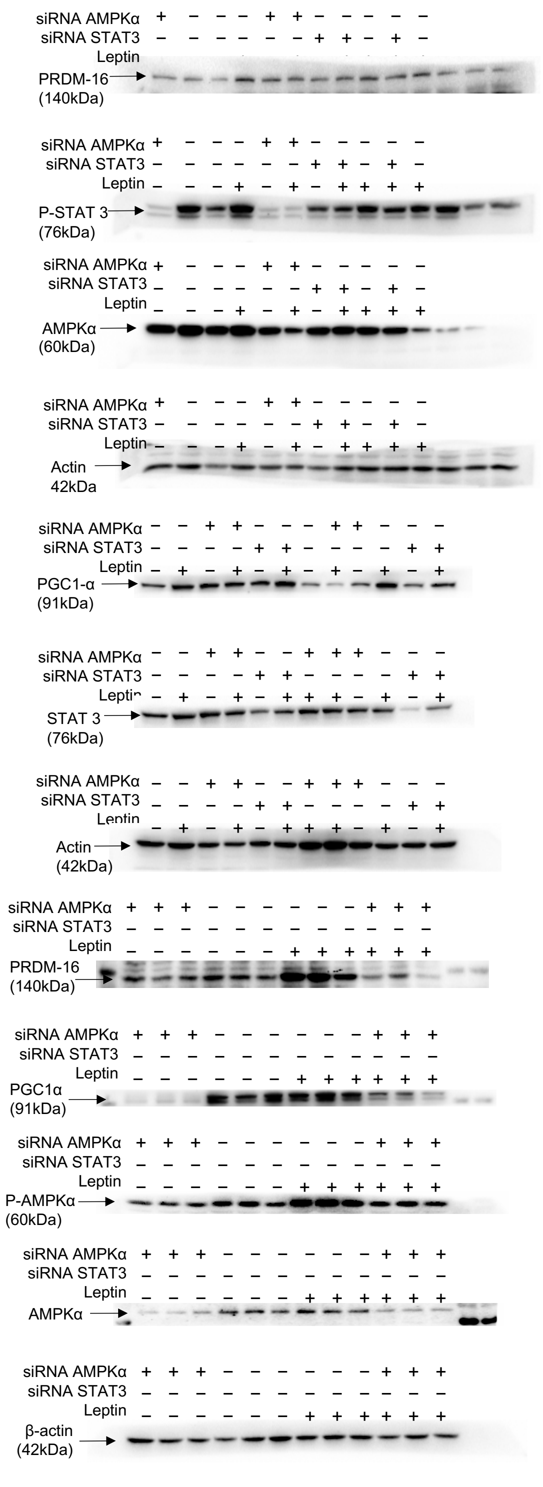

Fig.6E

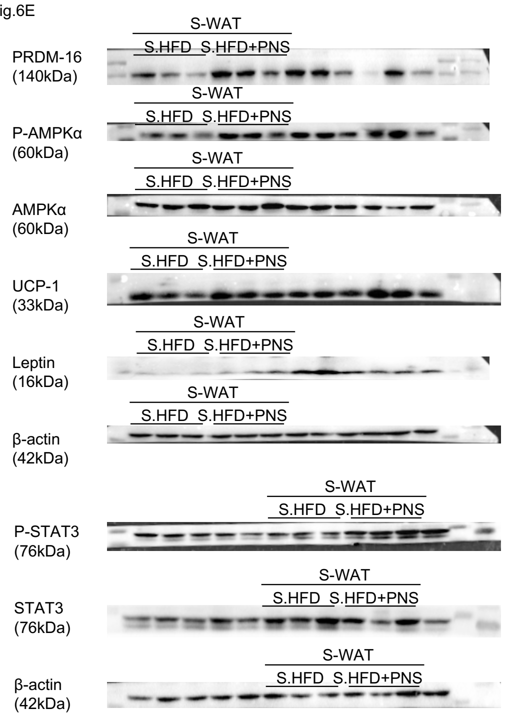

Fig.7G

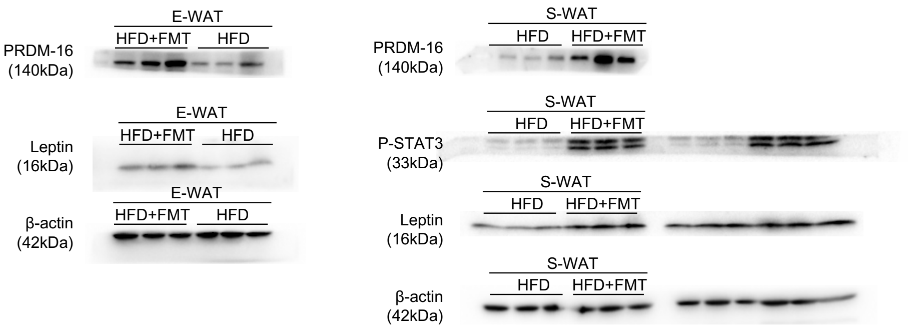

Figure S10| Original western blots from figures 2 to 7. The original, uncropped western blot membranes or film scans are shown. Figures 2G, 3B, 3F, 3G,4B, 4C,6E amd 7G included.

Supplementary Table 1. Primer sequences used for the quantitative PCR

Primer Name	Sequence (5' to 3')
Mouse UCP1 F	CCCGCTGGACACTGCC
Mouse UCP1 R	ACCTAATGGTACTGGAAGCCTGG
Mouse PGC1-α F	AACCACACCCACAGGATCAGA
Mouse PGC1-α R	CTCTTCGCTTTATTGCTCCATGA
Mouse Leptin F	GAGACCCCTGTGTCGGTTC
Mouse Leptin R	CTGCGTGTGTGAAATGTCATTG
Mouse Cd36 F	GCCAAGCTATTGCGACATGA
Mouse Cd36 R	TCTCAATGTCCGAGACTTTTCAAC
Mouse PRMD16 F	CCACCAGCGAGGACTTCAC
Mouse PRMD16 R	GGAGGACTCTCGTAGCTCGAA
Mouse Dio2 F	TGCGCTGTGTCTGGAACAG
Mouse Dio2 R	CTGGAATTGGGAGCATCTTCA
Mouse Adrb3 F	TCGACATGTTCCTCCACCAA
Mouse Adrb3 R	GATGGTCCAAGATGGTGCTT
Univeral 16S rRNA F	TCCTACGGGAGGCAGCAGCAGT
Univeral 16S rRNA R	GGACTACCAGGGTATCTAATCCTGTT
A. muciniphila F	CAGCACGTGAAGGTGGGGAC
A. muciniphila R	CCTTGCGGTTGGCTTCAGAT
P. distasonis F	TGCCTATCAGAGGGGGATAAC
P. distasonis R	GCAAATATTCCCATGCGGGAT

Supplementary Table S2. List of Antibodies

Antibodies	Source	Catalogue #
Rabbit polyclonal, AMPKα	Cell Signaling Technology	#2603
Mouse monoclonal,STAT3	Cell Signaling Technology	#9139
Rabbit polyclonal,β-actin	Cell Signaling Technology	#4967
Rabbit polyclonal, phospho-AMPKα	Cell Signaling Technology	#2535S
Rabbit polyclonal, phospho-STAT3	Cell Signaling Technology	#9145
Mouse monoclonal,UCP-1	R&D system	MAB6158
Rabbit polyclonal, PRDM16	Abcam	ab106410
Rabbit polyclonal,leptin	Abcam	ab9749
PGC1α (NBP1-04676)	Novus Biologicals	NBP1-04676
